# Tipping the Balance: Robustness of Tip Cell Selection, Migration and Fusion in Angiogenesis

**DOI:** 10.1371/journal.pcbi.1000549

**Published:** 2009-10-30

**Authors:** Katie Bentley, Giovanni Mariggi, Holger Gerhardt, Paul A. Bates

**Affiliations:** 1 Biomolecular Modelling Laboratory, Cancer Research UK London Research Institute, London, United Kingdom; 2 Vascular Biology Laboratory, Cancer Research UK London Research Institute, London, United Kingdom; UT Southwestern Medical Center, United States of America

## Abstract

Vascular abnormalities contribute to many diseases such as cancer and diabetic retinopathy. In angiogenesis new blood vessels, headed by a migrating tip cell, sprout from pre-existing vessels in response to signals, e.g., vascular endothelial growth factor (VEGF). Tip cells meet and fuse (anastomosis) to form blood-flow supporting loops. Tip cell selection is achieved by Dll4-Notch mediated lateral inhibition resulting, under normal conditions, in an interleaved arrangement of tip and non-migrating stalk cells. Previously, we showed that the increased VEGF levels found in many diseases can cause the delayed negative feedback of lateral inhibition to produce abnormal oscillations of tip/stalk cell fates. Here we describe the development and implementation of a novel physics-based hierarchical agent model, tightly coupled to *in vivo* data, to explore the system dynamics as perpetual lateral inhibition combines with tip cell migration and fusion. We explore the tipping point between normal and abnormal sprouting as VEGF increases. A novel filopodia-adhesion driven migration mechanism is presented and validated against *in vivo* data. Due to the unique feature of ongoing lateral inhibition, ‘stabilised’ tip/stalk cell patterns show sensitivity to the formation of new cell-cell junctions during fusion: we predict cell fates can reverse. The fusing tip cells become inhibited and neighbouring stalk cells flip fate, recursively providing new tip cells. Junction size emerges as a key factor in establishing a stable tip/stalk pattern. Cell-cell junctions elongate as tip cells migrate, which is shown to provide positive feedback to lateral inhibition, causing it to be more susceptible to pathological oscillations. Importantly, down-regulation of the migratory pathway alone is shown to be sufficient to rescue the sprouting system from oscillation and restore stability. Thus we suggest the use of migration inhibitors as therapeutic agents for vascular normalisation in cancer.

## Author Summary

Abnormal vasculature exacerbates many diseases such as cancer and diabetic retinopathy. In angiogenesis new blood vessels, headed by a migrating tip cell, sprout from pre-existing vessels in response to chemical signals. The signals are released from newly oxygen deficient tissue. The signals are known to be different in disease and are thought to cause the process of angiogenesis to progress abnormally, though the reasons for this remain unclear. Normalisation of angiogenesis has great potential as a therapeutic strategy; it has been shown to reduce metastasis and improve drug delivery in tumours. Here we focus on the behaviours of three inter-related initial angiogenic pathways associated with changes in tissue signal conditions, utilising both *in silico* and *in vivo* approaches. By the construction and implementation of a novel computational model for cell motility and signal processing we present a new theory on why angiogenesis exhibits such sensitivity to signal changes and show that the behaviour in disease is surprisingly more robust than normal functioning. This we attribute to the positive feedback of cell migration reinforcing abnormal oscillations in cell fate selection. We make the unique prediction that normalisation could be achieved by reducing cell migration alone.

## Introduction

Sprouting angiogenesis [1] is integral to a plethora of normal and pathological biological processes, such as embryonic development, wound healing and cancer. Sprouts are headed by migrating tip cells, which exert pulling forces on their neighbouring stalk cells. Tip cells are guided by, amongst other signals, external VEGF gradients released by nearby oxygen deficient tissue [2]. Tip cells meet and fuse, forming blood vessel loops (anastomosis) which can support flow, though the full mechanism remains unclear [3]. A dense vascular network builds up, which is later pruned by remodelling processes related to flow [4].

Under pathological conditions, such as cancer and diabetic retinopathy, overly high VEGF concentrations are observed, resulting in aberrant sprouting and a tortuous, leaky network with poor perfusion [5],[6]. Normalisation of blood vessel development in disease has important therapeutic potential. Improving vascularisation and blood flow in cancer can reduce the occurrence of metastatic cells by lowering tumour hypoxia and mutation rate [6]. Normalisation also improves drug delivery by creating better supply lines to the tumour cells [6]. Our overall aim is to first use a systems approach to understand how changes in VEGF result in a switch between normal and abnormal tip cell selection, sprouting and fusion and then predict methods to tip the balance back to normal angiogenesis in disease. Our main approach is computational modelling, which has been highlighted as essential when the dynamics of a process are so intricate and multi-faceted that predicting the outcome of perturbations is no longer intuitive [7]. However, whilst the model we present here is an adaptable, modular, generic computer algorithm, easily transferable to the investigation of cell motility/signalling dynamics in a diverse range of biological applications, it is highly important that models maintain focus if we are to answer specific questions in a chosen biological domain. Therefore, we keep our parsimonious *in silico* model closely tied to quantitative *in vivo* angiogenesis imaging data throughout the paper; an approach recently highlighted as imperative for understanding complex developmental systems [8].

Angiogenesis has previously been modelled using a variety of approaches including cellular automata and continuum models [9]–[14]. Individual-based approaches, such as agent-based modelling used here, have been highlighted as more appropriate over continuum methods for sprouting simulations, which involve low numbers of cells [15]. Other models considering the early stages of angiogenic sprouting have focussed on endothelial cells themselves releasing diffusible attractants and inhibitors [15]–[18]. However, recent evidence shows Dll4-Notch juxtacrine signalling drives sprout initialisation and external gradients of attractants released by oxygen deficient tissue guides the sprouting process [2],[19]. Recently a simple binary model of tip cell selection was used in a sprouting model [20], but did not involve the essential ingredient of dynamic Dll4-Notch lateral inhibition and instead allocated tip cell status based on exceeding a single threshold of environmental VEGF.

In our work, by modelling the full interconnected pathways and protein interactions underlying tip cell selection and migration, we elucidate the combined, system level effects of ongoing lateral inhibition with migration, fusion and increasing VEGF concentrations. The central, novel feature of this model is that tip/stalk cell fates are continually in flux, due to the realistic modelling of ongoing Dll4/Notch lateral inhibition, known to control tip cell selection [19]. Fates are not permanently fixed, as in other models [16],[20]. This yields significant new insights into the subtle dynamics, sensitivities and recursive nature of the process. Moreover, to the Authors knowledge, this is the first computer model to consider physical mechanisms of filopodia (long, thin, dynamic actin-based membrane protrusions) led migration of adhered endothelial cells in vessels, the complex morphological changes that occur in cell shape and junction size during sprouting/fusion and the spatial feedback this gives to the ongoing signalling networks.

**Figure 1:**
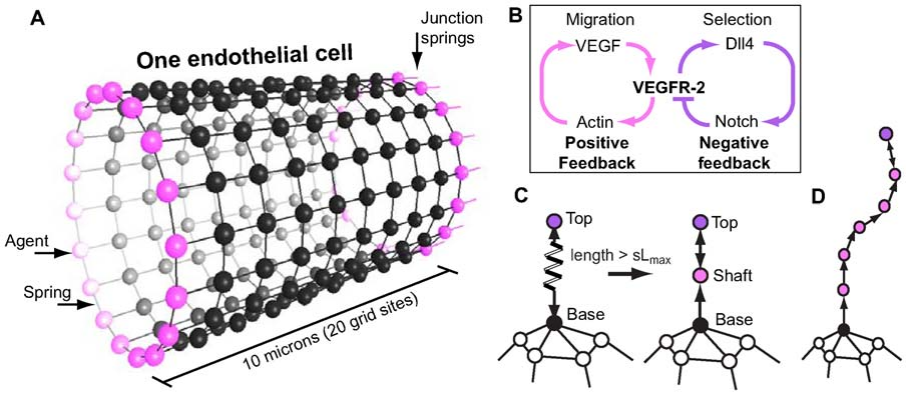
Overview of the spring-agent model. (A) An endothelial cell is represented by a cylindrical, single layer square lattice mesh of agents, connected by springs, the physical properties for which follow Hookes Law. At either end (adjacent cells not shown) specialised junction springs and agents (pink) connect the cell to its neighbour cells along the vessel. (B) The underlying pathways modelled. VEGF activates VEGFR-2 receptors leading to both upregulation of Dll4 ligands and activation of actin polymerisation. Actin-based filopodia create positive feedback by increasing the cells surface area and aiding migration up the VEGF gradient, resulting in increased VEGF exposure. Dll4 binds to Notch receptors on neighbouring cells generating negative feedback by down regulating VEGFR-2 receptors. (C) To grow a filopodia a new agent and spring are created, see text for more details. When a filopodia spring exceeds the threshold length (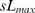) a new node is inserted half way along the spring. It is given state ‘shaft’ and a focal adhesion. The original spring/s are deleted and new ones are created to reconnect the agents. Upon insertion of new nodes a spring connecting the top node back to the next node is also created. This is needed if the filopodia top node begins to retract. (D) As the filopodia extends shaft adhesions are created at regular intervals.

An agent-based model is utilised here as it provides a simple, effective methodology for capturing the distributed hierachical and morphological aspects of the multiscale sprouting process. Physical tension in the membrane is modelled by the inclusion of springs following Hookes law, connecting the memAgents, see Fig 1(A), facilitating realistic modelling of filopodia-led cell migration. Springs have been employed to great effect in a number of other biological models, such as in nematode sperm migration [21], where springs simulate internal actin filament cross-connections driving the lamellapodia of a single cell. Another example uses springs to mimic forces *between* cells, of migrating enterocytes in the intestine [22] and epithelial cells dividing and sorting in the intestinal crypt [23]. However, here we make the significant extension that springs model both the internal actin-based cortical tension and filopodia driven migration in single cells, but also the higher, cell-cell level interactions of multiple, adhered cells.

Fig. 1(B) shows the pathways we include in the model in a simplified manner: the actin driven migration pathway and the Notch-driven selection pathway; both require activation of the receptor VEGFR-2. Migration is known to occur through the kinases p38 and PI3K, leading to actin polymerisation and filopodia-led migratory behaviour [24]–[26]. It can be seen as a positive feedback loop as further actin-driven movement forward will increase the input signal of VEGF. The Notch pathway is a negative feedback loop: VEGF binds to VEGFR-2 receptors on the endothelial cells, which up-regulates expression of the ligand Dll4 [27]. Dll4 binds to Notch receptors on neighbouring cells via juxtacrine signalling and results in the down-regulation of VEGFR-2 receptors in the neighbour cell [28]–[30]. Under normal conditions this lateral inhibition has been shown, *in silico* and *in vivo*, to generate an evenly scattered arrangement of tip cells separated by not more than two stalk cells (termed a ‘salt and pepper pattern’). Moreover, under pathological conditions we previously predicted that cell fates can oscillate [31]. This is consistent with observations made in other systems, where negative feedback combined with high input signals and delays (caused by rates of gene expression and protein transport) produces oscillations [32].

Three separate investigations were performed with the model leading to the following main conclusions/predictions. First, analysis of emergent behaviour observed in the model led to the prediction that, under normal VEGF conditions, tip cell fusion locally disrupts stabilised tip/stalk selection patterns, by altering the network of interconnected cells, and causes a local flip in tip/stalk fates; the new junction formed when tip cells fuse creates a new influence on the ongoing lateral inhibition. In contrast, oscillations in tip/stalk fate, occurring in high VEGF, are shown to be robust to anastomosis events. Secondly we predict tip cell migration alone has a destabilising effect on Dll4/Notch selection as it causes lengthening and distortion of cell-cell junction sizes. Junction size changes affect the strength of Dll4/Notch signalling between different neighbours. This is shown to contribute to the switch between normal and pathological angiogenesis. We go on to make a novel prediction for therapeutic intervention from this study: reducing migration can normalise sprouting in pathologically high VEGF environments. Finally, validation of a novel filopodia-adhesion driven mechanism for migration, based on the filopodia-adhesion driven migration of neuronal growth cones described in [33], is presented by comparing our *in silico* model with *in vivo* data from zebrafish vascular development.

## Model

The novel Spring-Agent model presented here is based on the agent-based model described in [31] with the significant extension that here physical cortical tension is included, by incorporating springs following Hookes law. Here we will limit description to focus on the new aspect of spring inclusion only, see Supporting Information (text S1) for a brief overview of the original model and for full details see [31].

The Spring-Agent model is hierarchical. A cell ‘agent’ is comprised of smaller agents, which represent sections of the cell membrane (termed ‘memAgents’). Springs connect these memAgents together conferring tension to the cell's periphery, see Fig. 1(A). MemAgent's then act autonomously to drive local membrane responses of the cell to local environmental changes. Therefore, the overall behavioural responses of a cell are emergent from the lower level interactions of its many memAgents; however, cell agents do act as a repository for total protein activation information across all memAgents and can use these totals to affect the global, cellular protein levels at the next timestep (genetic regulation).

The springs represent tension found in the underlying actin cortex below the membrane, rather than in the membrane itself. Membrane is known to show poor elasticity, tearing at 4% strain and instead has a rapid addition/subtraction mechanism to maintain the required membrane size [34]. The model therefore also incorporates a dynamic mechanism of memAgent addition/subtraction to maintain a smooth surface as the cell changes shape, see surface coverage method described below. Further validation that the tension generated by the springs gives realistic cell curvature during migration, is given in Supporting Information (text S2, video S5, fig S1). The springs allow realistic modelling of actin driven filopodia extension by cells, migration and eventual fusion by forming new cell-cell junctions between migrating cells when they meet.

In order to accurately model local membrane movements generated by small increments in spring length, memAgents were simulated in three-dimensional continuous space. However, to allow efficient assessment of each memAgent's local environment, memAgents were periodically snapped to a three-dimensional gridded lattice. Due to this, more than one memAgent could exist in one lattice site. The Moore neighbourhood, which includes the adjacent and diagonal lattice neighbours, was used for local rule updating. In our approach the embodied problem is considered, rather than modelling migration in isolation, it is the interaction of cells *and environment*, that is modelled. This led us to use a standardised square lattice grid representation of space across the cells and the environment; therefore, for simplicity, the cell's springs were also modelled as a square mesh. Lattice grid sites also contained information about environmental factors such as VEGF, as will be described.

**Figure 2:**
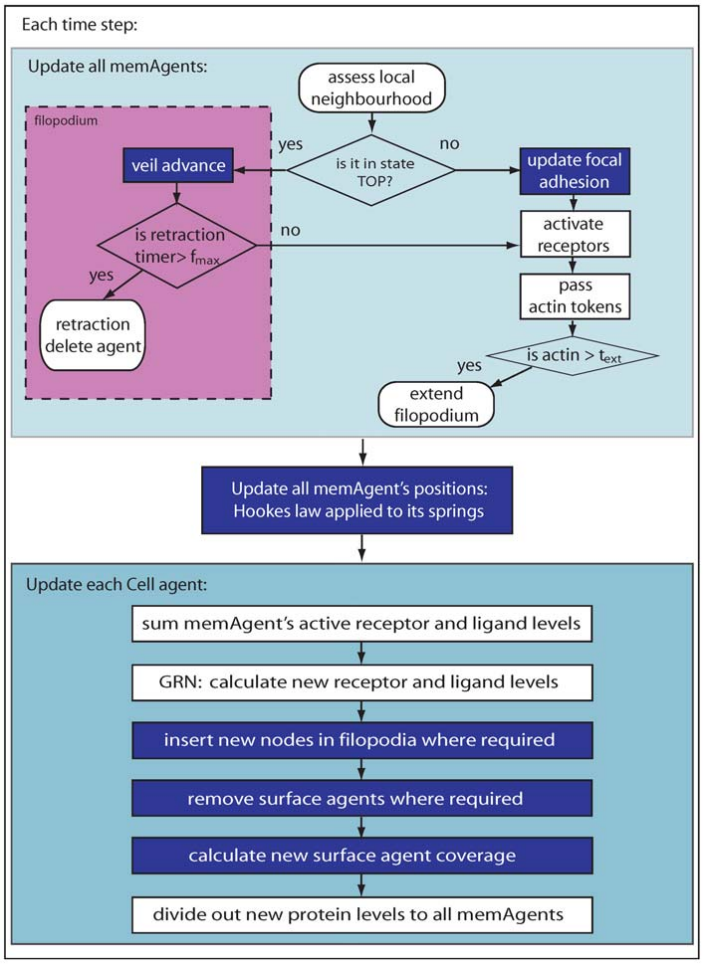
Flow diagram of the model. Blue boxes indicate spring procedures. First, all membrane agents are updated in asynchronous random order, then, based on spring length adjustments, their positions are updated. Second, each cell agent updates its total protein levels across all its associated membrane agents, inserts new nodes where required and recalculates its surface agent coverage.

To initialise the model, a hollow cylindrical vessel, 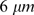 in diameter (based on average capillary dimensions [35],[36]) comprising a number of 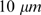 wide connected cells was placed at the bottom of a coordinate system, along the 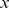 axis, with an arrangement of VEGF in the environment. To eliminate bias, periodic boundary conditions were employed, joining the two ends of the vessel into a continuous ring. Only the ablumenal cell surface was simulated for simplicity; there is no cell interior. The model was implemented in C++ using openGL graphics on a standard workstation. See the flow diagram in Fig. 2 for a complete overview of the simulation procedure; each new module, shown in blue, is described below followed by a full summary of the simulation procedure. See Table 1 for all model parameters, including initial positions and dimensions. One grid site represents a cube, with sides of length 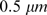.

**Table 1:** Model dimensions and parameters.

Parameter	Definition	Setting	Reference
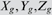	lattice width, length, depth	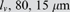	model specific
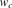	width of EC agents	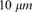	[35]
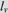	vessel length	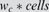	simulation specific
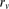	radius of vessel	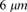	[35]
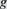	gap between vessel and lattice boundary	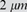	model specific
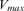	max VEGFR-2 receptors	31740	calculated from [61]
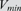	min VEGFR-2 receptors	690	estimated
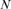	Notch receptors	10,000	estimated
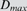	max Dll4	10,000	estimated from [27]
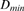	min Dll4 ligands	0	estimated
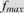	max time before memAgent collapses filopodia	10 timesteps	estimated from [62]
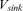	proportion of VEGF left by VEGFR-1	0.11	estimated
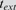	Actin tokens required for filopodia extension	3	estimated
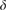	Dll4 expression change due to VEGFR-2	2	evaluated in [31]
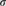	VEGFR-2 expression change due to Notch	15	evaluated in [31]
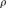	significance range for pattern stability	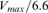	model specific
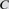	strength of VEGFR-2 actin activation signal	2	evaluated in [31]
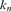	Mesh spring constant	0.05	optimised
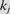	Junction spring constant	0.4	optimised
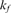	Filopodia advancing spring constant	0.7	optimised
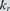	Filopodia retraction spring constant	0.95	optimised
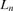	Mesh ideal spring length	1.0	optimised
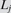	Junction ideal spring length	0.5	optimised
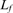	filopodia ideal spring length	0.1	optimised
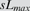	filopodia shaft spring length	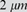	[33]
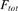	maximum total length of filopodia on a cell	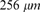	estimated

### Agent states

MemAgents have two states, which define their overall type and determine the behaviours they can choose to implement: 1) their physical state and 2) their filopodia-related location. The physical state is set as either: node, spring or surface. Node agents have springs connecting them to other node agents, forming the main mesh, and are part of the physical structure of the membrane. Spring and surface agents do not apply forces. They are created and deleted on-the-fly, sitting upon the mesh, to maintain a continuous membrane surface as the mesh changes shape, they are explained in the next section. A memAgent's physical type remains set for its lifetime.

The filopodia state is set as either: none, base, shaft or top, see Fig. 1(C). All memAgents are initialised in state ‘none’ (not in a filopodia). Each memAgent is allocated a number of VEGFR-2, Notch and Dll4 by its cell agent at each time step, as described in [31] and text S1. It then changes its filopodia state based on the accumulation of actin, accrued by local VEGF activating its VEGFR-2 receptors. See Tables 2, 3 and 4 for the attributes and initial settings of each agent type in the system.

**Table 2:** Initial Cell agent internal knowledge settings.

Attribute	Description	Initial setting	Range
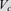	level of VEGFR-2	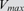	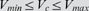
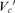	level of active VEGFR-2	0	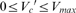
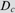	level of Dll4	0	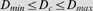
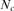	level of Notch	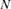	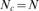
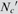	level of active Notch	0	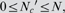
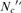	effective active Notch	0	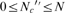
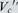	effective active VEGFR-2	0	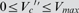
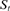	stability score at timestep 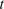	0	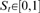
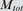	cell's total number of memAgents	949	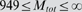

**Table 3:** Initial memAgent internal knowledge settings.

Attribute	Description	Initial Setting
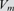	level of VEGFR-2	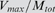
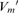	level of active VEGFR-2	0
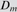	level of Dll4	0
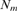	level of Notch (if 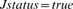 )	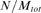
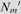	level of active Notch	0
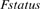	indicates memAgent's location in a filopodia	none
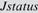	indicates if memAgent resides at a cell-cell junction	true/false
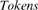	no. of actin tokens	0
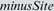	previous memAgent in filopodia	NULL
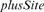	memAgent ahead in filopodia	NULL
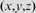	position, stored in real and integer form	
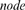	whether its a node or surface agent	true/false
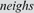	list of node neighbours connected by springs	memAgent addresses
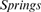	list of connected springs	spring addresses
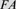	if a node, whether it has a focal adhesion	true/false

**Table 4:** Spring object internal knowledge settings.

Attribute	Description	Setting
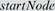	node spring originates from	memAgent address
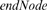	node spring culminates at	memAgent address
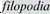	is it in a filopodia	true/false
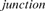	is it a junction spring between cells	true/false
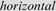	is it classed as horizontal or vertical	true/false
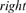	is it classed as left or right	true/false
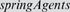	list of spring coverage agents belonging to this spring	memAgent addresses

### Surface and spring agent coverage

As the square mesh representing the cell expands and stretches, the space between nodes increases. In order to represent the continuous nature of the cell surface, when snapped to the 3D gridded lattice, a voxelisation algorithm was used. Without it holes would occur as nodes get stretched further apart by migration forces, leaving apparent gaps in the mesh. To cover the surface, the space between each four-node square of the spring-agent mesh is divided into two triangles. The triangles are then voxelised, creating a new memAgent at each grid site that the triangle plane passes through, see Supporting Information for details of the voxelisation algorithm (text S3, fig S2, S3, S4, S5). The new memAgents created to complete the surface are allocated the physical state ‘surface’ as opposed to ‘node’. Only node agents are allowed to extend filopodia - as these are the only agents which can convey forces through the mesh. The ‘surface’ agents instead sit on top of the mesh, associated with a particular surface triangle. They pass actin they accrue from their receptor activation to the nearest node agent of their triangle.

For continuous coverage of filopodia springs, a new memAgent is created in every grid site that the filopodia passes through. These ‘spring’ agents pass any actin they accrue upwards towards the top node, which can then use them to extend. When springs in a filopodium are deleted and new ones created, e.g. in shaft node addition/deletion (described below), the spring agents associated with the deleted springs are reassigned to the new springs. This maintains continuity in actin token possession along the filopodia.

### Spring forces

All node memAgents that do not have an adhesion to the substrate, update their position each time step, according to the forces being applied to them by their springs. MemAgents with an adhesion remain stationary, anchored to their current position. The force 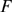 on a node 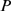 of its 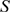 springs is given by:
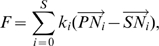
(1)where 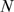 is the vector position of the neighbour node at the other end of the spring and 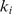 is the specific spring constant for that type of spring. There are three types of spring in the model, normal mesh, filopodia and junction springs. See Table 1 for the specific spring parameters of each type. Spring constants were estimated by matching morphological curvature of tip and stalk cells with confocal images. If 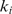 is too high, then the curve of the cell is too sharp and straight, thus callibrating these constants against images yielded realistic curvature and migration speeds in the model. A full sensitivity analysis of these spring constants was also performed, details are given in the results section. SN is given by:
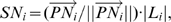
(2)where 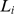 is the natural rest spring length for that type of spring. The new position of node agent 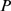 is given by 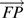. However, since biological membranes can contract without springing back out, springs in the model do not generate a force when the spring length goes below the set ideal length (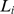). Junctions between cells are represented by a specific type of ‘junction spring’. To keep junctions between cells tight, junction springs have a stronger spring constant, see Table 1.

### Filopodia

Biological cells are able to extend a number of different types of protrusion [37]. Endothelial tip cells almost exclusively favour filopodia, which contain long parallel bundles of actin filaments [2], these help the cell to navigate [38]. We base the filopodia dynamics in the model principally on insights from sensory growth cone studies reported in [33], where filopodia are shown to have three morphologically distinct regions: basal, shaft and tip (hereafter tip is referred to as top to avoid confusion with tip cells). Each region has its own type of adhesion with distinct functionality. Basal adhesions are critical to filopodia initiation, shaft adhesions inhibit veil advance (migratory advance of the cell body) and the top adhesions can signal the release of all shaft adhesions when a certain environmental stimulus is found [33]. This release allows the cell body to quickly advance in the optimal direction.

#### Extension

Initial outgrowth of a filopodium is modelled as follows. If a node agent in state ‘none’ (not in a filopodia) has stochastically acquired new actin on the current timestep (see Text S1 for full equations), and has a sufficient store of actin (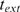), it can extend a new filopodium. In doing so, it changes to ‘base’ state and creates a new node agent in state ‘top’ ahead of it. See [31] for details of the actin system. The two agents are then connected by a spring in both directions. The newly created agent at the top is allocated an adhesion, so unless filopodia retraction is implemented (described below) it will not be pulled back towards the cell by the springs, and instead it pulls the non-adhered base towards it.

To extend an already growing filopodia, a top state agent with sufficient actin simply moves itself to a neighbouring grid site. This fits with reported rates of filopodia extension, 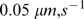 [38],[39]. The top agent had a 90% chance of moving to the grid neighbour with the highest VEGF concentration, otherwise a random grid site neighbour was chosen, provided that the current spring length increased.

Filopodia can only extend if the maximum, total filopodia length for that cell has not been reached, see Table 1. This maximum did not dictate a maximum filopodium length, but rather a maximum amount of actin available, expressed in terms of total polymer length across all filopodia. This lead to the realistic case where some cells have one or two very long filopodia, while others have many short filopodia.

#### Shaft adhesions and new nodes

**Figure 3:**
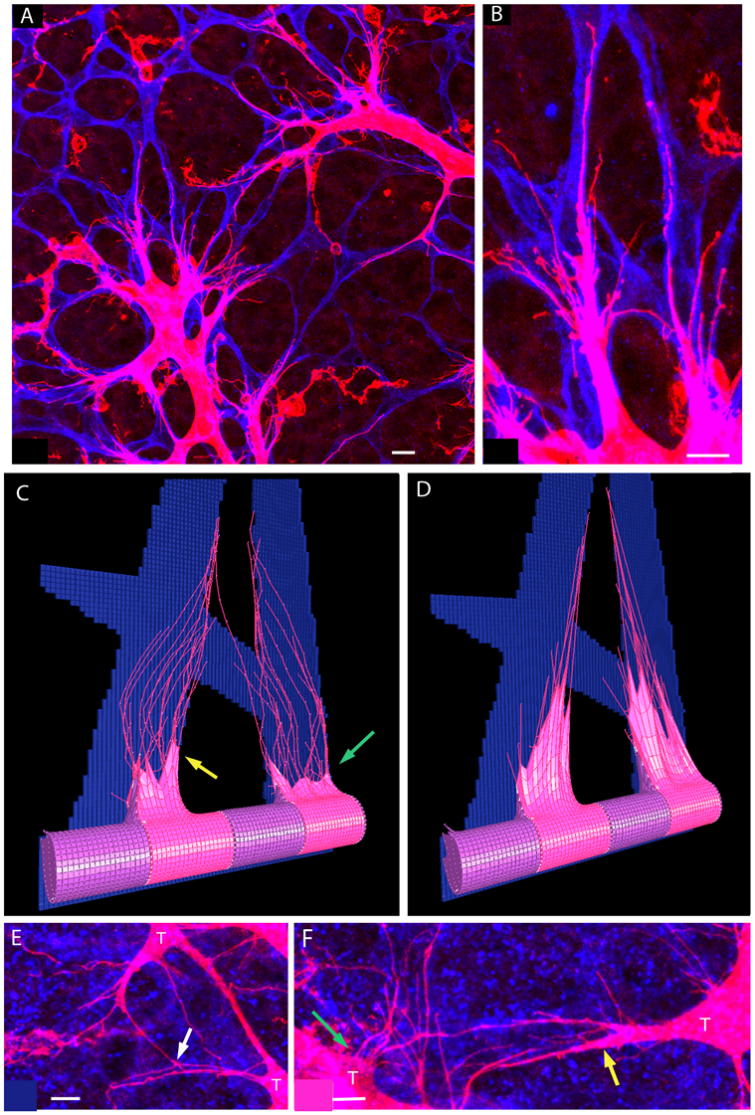
*In vivo* and *in silico* retinal sprouting, filopodia and astrocytes. (A) A typical confocal image of the developing mouse retinal vasculature showing endothelial cells with numerous long thin, curved filopodia (pink; isolectin B4 staining) and astrocytes (blue: 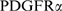 staining). (B) A zoomed in section of (A) used as a basis for the simulations, both 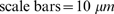. Two tip cells can be seen migrating up the prongs of an ‘A’ shaped astrocyte region. (C) The model with matching astrocyte environment. The two tip cells head towards the local point source of VEGF above. Colour indicates VEGFR-2 level (high - pink (tip cell), low - purple (stalk cell)). Junction springs are shown in white. Shaft adhesions on inserted nodes along filopodia facilitate realistic local curvature of filopodia. Shaft adhesions also inhibit veil advance (green arrows), giving more realistic cell morphology. Yellow arrow shows a filopodia where contact with a neighbour cell's filopodia has triggered veil advance. (D) Switching the shaft node mechanism off yields unrealistic shape. (E) Confocal image in the retina of a filopodia contact, which may be where signalling takes place to trigger veil advance. (F) Confocal image in the retina showing apparent inhibition of veil advance by filopodia (green arrow) and a filopodia which appears to have triggered veil advance (yellow arrow). Pink: isolectin B4 staining of endothelial cells and blue: astrocytes, 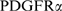 staining. Tip cells labelled T, 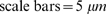.

To implement shaft adhesions, a new node is created at the midpoint of a filopodium spring when it exceeds a threshold length (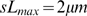, fitting with data in [33]). See Fig. 1(C). Thus filopodia are actually a connected chain of short springs and nodes. This benefited the model in three ways: 1) filopodia can bend and curve round corners, as seen in images, e.g Fig. 3(A,B); 2) the new adhered nodes inhibit migration; and 3) filopodia can retract in discrete steps, as each spring retracts, fitting with observational data [40]. Shaft adhesions have been shown to form in this *de novo* way along the filopodium, rather than being top adhesions retained as the filopodia elongates [33].

#### Veil advance

Filopodia shaft adhesions are inhibitory to cell migration until released [41]; Once released the cell body or ‘veil’ can advance along the filopodium[33]. To implement the signalled release of shaft adhesions an agent communication method is used. If a ‘top’ memAgent detects, in its local neighbourhood, a memAgent from a different cell's filopodia, it can trigger the veil advance behaviour; the top memAgent passes a message back down its filopodium to the next shaft node, if one exists. The message tells it to release its adhesion, set the springs status to ‘veil advancing’ and pass the same message down to the next node and so on until the base node is reached. A spring with ‘veil advancing’ status can exceed the threshold length for creating a shaft node, as inhibiting shaft adhesions no longer need to be inserted.

During veil advance a filopodium's springs pull their unadhered shaft nodes towards the top. Inevitably the shaft nodes collect at the top, thus, a clear up function was implemented. When the distance between two shaft nodes 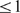 grid site length, the shaft node closest to the top was deleted, along with its associated springs. The node below was then connected by a new spring to the node above.

#### Retraction

Filopodia are dynamic structures, they extend and retract. This is implemented in the model as follows. The filopodia's top memAgent has a counter incremented every consecutive timestep that the agent fails to extend. Once this counter reaches a threshold (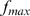) the agent changes its state to ‘retracting’ and releases its adhesion. Its spring then pulls it back towards the cell body. Following the principle of the node clear-up function described above, if the distance between the top node and its connected neighbour node is less than or equal to one grid site then the top node agent, and its associated springs, is deleted. If the connected neighbour below is: a) the base node, then it is set to ‘none’ state, completing retraction; or b) a stalk node then it becomes the new top node, with no adhesion, and retraction continues.

### Cell-cell fusion: anastomosis

As tip cells migrate, once the veil advance mechanism has been triggered, they meet other tip cells and ‘fuse’ forming a new junction, termed *anastomosis*. To implement new junction formation: if any two non-filopodia memAgents, from different tip cells, are located on adjacent grid sites, they change their status to ‘junction’ and create a new ‘junction’ spring between them. If either memAgent is a ‘surface’ agent, then the new junction spring is created from the nearest node of the surface triangle to which it belongs. If a new junction spring is created between two agents, then new junction springs are also created between all direct neighbour nodes in the mesh, with the corresponding node neighbours on the other tip cell, which helps zipper up the newly formed junction. To limit excessive junction springs, a new spring is only created if both nodes contain no other junction springs to the that cell.

### Environment

Figure 3(A) shows blood vessels in the retina sat upon the astrocytic network, a network of inter-connected star-shaped astrocyte cells. The 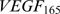 isoform is known to adhere to astrocytes rather than freely diffusing through interstitial space, as opposed to the 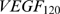 isoform [42]. In this work, only the 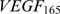 isoform is modelled. Two simple astrocyte patterns were used in the simulations. The first was based on a section of real retina, which biases the second and fourth cells to become tips, in a row of four cells, see Fig 3(B, C). This topology was used when observing system behaviour, and where selection dynamics and bias were not a concern. To implement this, the state ‘astrocyte’ was assigned to grid sites within a predefined ‘A shape’ pattern as seen in confocal image Fig 3(B). The second astrocyte pattern was a simple unbiased square latticework; all cells experience the same VEGF levels and the same layout of astrocytes ahead. To implement this the state ‘astrocyte’ was assigned to grid sites within a predefined pattern of interlacing strutts with equal dimensions, width 9, depth 4 grid sites, extending to the grid boundaries. MemAgents could not move into ‘astrocyte’ grid sites; however, filopodia, given their thin representative size, could co-exist in an astrocyte grid site.

VEGF adherence to the astrocytes was modelled by allocating a number of VEGF molecules to grid sites with astrocytes in their local neighbourhood, starting ahead of the vessel, (only for grid sites where their 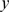 coordinate is greater than 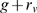, see Table 1). VEGF increased linearly in the 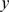 axis. However, this alone could only guide cells straight upwards, and in parallel. To force cells to meet, in simulations involving anastomosis, a short-range, local gradient from a point source of VEGF was overlayed. The use of local gradient ‘signposts’ fits with biological observations [2],[43]. Thus the amount of VEGF in each grid site was given by
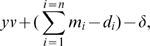
(3)where 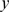 is the position of the grid site on the y axis, 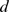 is the distance of the grid site from the point source, 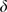 is a small random fluctuation, the value of which is randomly chosen from zero to 0.3 (the final VEGF level was not allowed to go below zero). 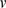 and 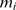 are the VEGF levels for the linear gradient and any point sources 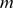 respectively. If the distance 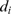 exceeds 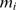 or is greater than the point sources maximum range 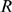, 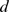 is reset to equal 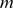 and thus no point source VEGF is allocated in that grid site. The ‘normal’ level of 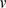 was set to 0.2 and 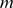 to 0.15 for all runs with point source VEGF. Point sources were placed at a distance of 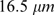 from the vessel, similar to the distance between fusing tip cells in the zebrafish and mouse retina data presented.

### Simulation overview

Referring back to Fig. 2 the whole model is simulated as follows. Within each timestep all memAgents are updated by: 1) assessing their nearest neighbours in the look up lattice-grid; 2) calculating their new receptor activation levels (VEGFR-2 is activated by local lattice site VEGF levels, Notch is activated by local Dll4 in memAgents from a neighbouring cell) then 3) deciding whether to implement any filopodia behaviours. Subsequently, once all memAgents have been updated, 4) the off-lattice positions of all node agents are recalculated; implementing Hookes law and applying to all springs.

Once all memAgents have updated, each cell agent: 1) calculates new total levels of active receptors and ligands (VEGFR-2 receptors are down-regulated by Notch activity, and Dll4 is up-regulated by VEGFR-2 activity, see text S1 for exact equations); 2) removes spring and surface agents and calculates new coverage of the mesh based on new node positions then; 3) Updated levels of receptors and ligands are equally distributed to all cell memAgents after a delay representing transcription/translation rates (Notch and Dll4 are localised to memAgents at junctions only). The process then repeats.

## Results

The three main investigations of this work are detailed in the following sections. First, the effects of tip cell fusion events on lateral inhibition, in different VEGF environments are presented, predicting tip/stalk fates can infact reverse. Then, the effects of migration and dynamic junction size on the stability of lateral inhibition as VEGF increases are explored. Normalisation of selection and sprouting in high VEGF environments is then achieved by exploring optimal junction arrangements and reducing the migration response. Finally, validation of the filopodia-adhesion driven mechanism of migration and fusion is given by comparing to quantitative experimental live imaging data and confocal microscopy.

### Dll4-Notch lateral inhibition during anastomosis causes cell fates to flip

Dll4-Notch lateral inhibition between endothelial cells is responsible for tip cell selection. However, lateral inhibition is an ongoing process, continuing throughout angiogenesis. The interaction between ongoing lateral inhibition and the further stages of angiogenesis, migration and anastomosis, have not yet been investigated *in vivo*, *in vitro* and not least *in silico*. An interesting, unexpected, emergent property of the system was observed in simulations when migration, anastomosis and lateral inhibition occur concurrently. The formation of a new junction between fusing tip cells during anastomosis provides a new border where the two tip cells now battle, via Dll4/Notch signalling, to inhibit each other. This causes a destabilisation of the current tip/stalk pattern and results in one of the fusing tip cells being inhibited, see video S2 in Supporting Information. We wanted to investigate whether this emergent property could cause a destabilisation along the entire vessel, and whether different starting selection patterns and VEGF concentrations would have an effect.

**Figure 4:**
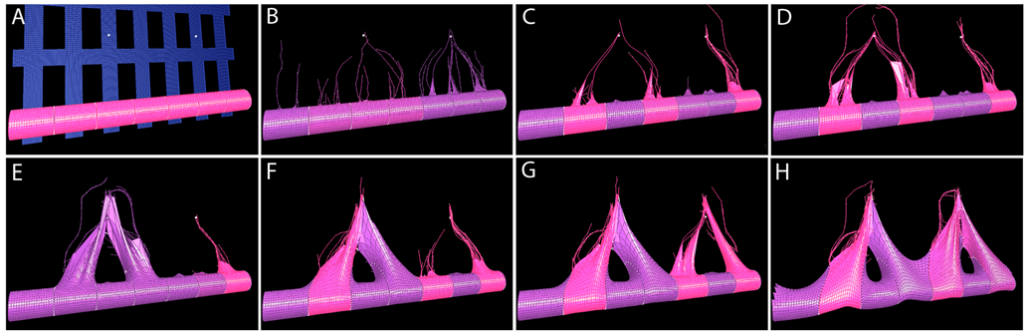
Simulation showing stalk cells flip fate upon anastomosis. Screenshots of a simulation with seven cells on a square astrocyte lattice (blue) with two local VEGF point sources (white dots). Cell colour indicates VEGFR-2 receptor levels, pink - high (tip cells), purple - low (stalk cells). (A–D) initial tip/stalk pattern is selected by Dll4-Notch lateral inhibition. (E) fusion of two tip cells leads to disruption of the stable tip/stalk pattern, as they become inhibited. (F) a new stable tip/stalk pattern is reached. Inhibition of a fused tip cell causes the neighbouring stalk cell to ‘flip fate’ and form a second vessel loop as seen in (G,H). It can be seen from (D) that the second loop would not be possible without the flip in cell fates.

**Figure 5:**
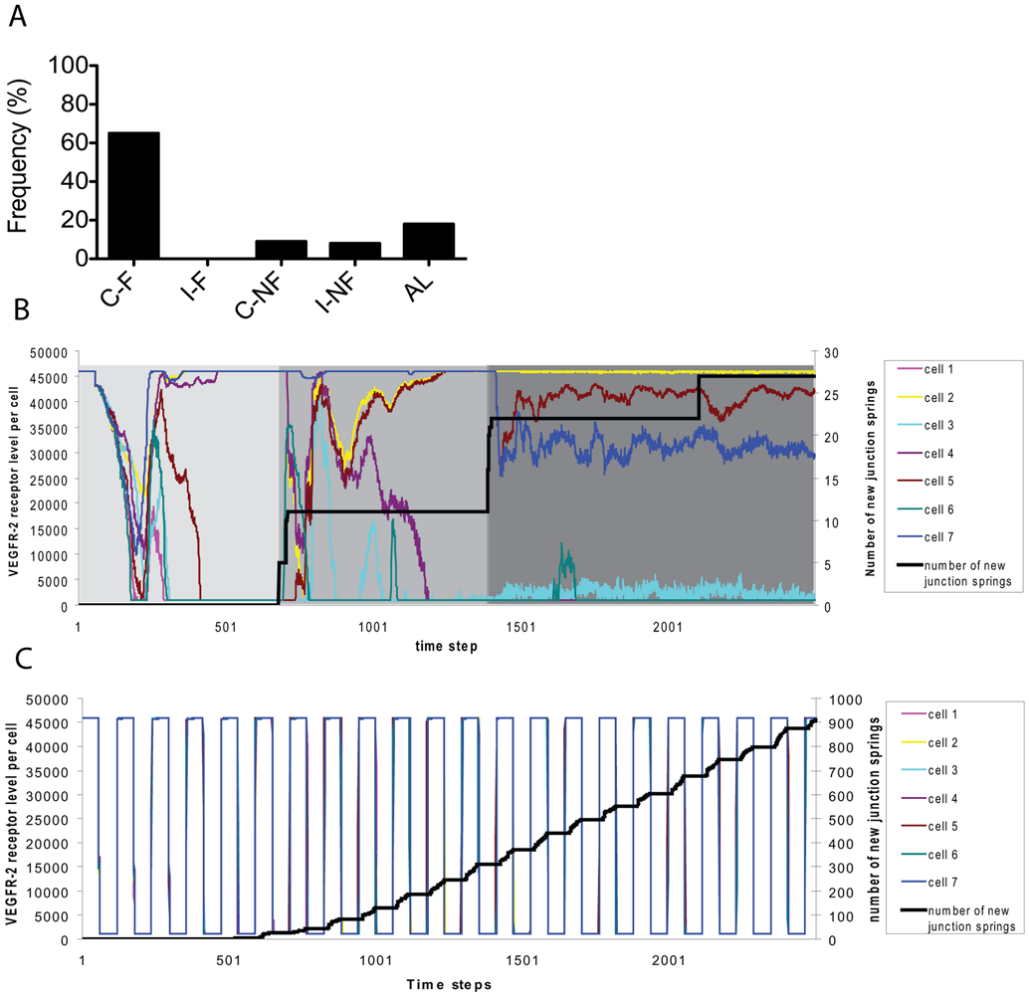
Graphs showing normal patterning is sensitive to fusion whereas oscillation is robust. (A) Frequency of one hundred events where cell fate, of one stalk cell to a tip cell, flips (F) or does not flip (NF) given which tip cell has been inhibted by the first anastomosis event - correct cell (C), the tip cell next to two adjacent stalk cells, or incorrect cell (I), or instead the first fusion event was between adjacent cells (AL). (B) Cell VEGFR-2 levels over one run in normal VEGF. The light grey region indicates the first selection of tip cells into a stable pattern. This pattern is disrupted by the generation of new junction springs during the first anastomosis event (black line). A new pattern then stabilises (medium grey region). Similarly, the dark grey region indicates the third phase of tip cell selection after the second anastomosis event. In the final phase, due to the simplicity of this particular simulation, the current tip cells become inhibited but no new reversals of fate occur. However, cell division, and extra VEGF point sources further into the environment, would cause this punctuated instability of cell fates to continue. (C) In 10 times the VEGF concentration a marked difference is observed in system behaviour. In high VEGF all cells synchronously oscillate between tip and stalk cell fate, unaffected by anastomosis events. New intercellular junctions are formed at a nearly continuous rate, due to all cells moving forward at once, leading to fusion along adjacent tip cells aswell as between three and four non-adjacent tip cells.

A vessel containing seven cells was initialised with two point sources of VEGF placed such that they may induce two loops, see Fig. 4. However, the number of cells in the vessel preclude more than one loop forming initially; only two or three tips can be stably selected from seven cells. Thus if two loops form a flip must have occurred in a stalk cell, caused by destabilisation from anastomosis of the first loop. To allow time for two loops to form, the simulation was run for a maximum of 8000 timesteps. See video S3 for a typical simulation run. It was found that only two of all the possible initial tip/stalk patterns could generate a flip in a fate and produce two loops instead of one: S T S T S S T and S T S S T S T. This is because they contain an adjacent set of stalk cells. If the tip cell inhibited during anastomosis is next to the adjacent stalk cells a row of four adjacent stalk cells is produced. This allows one stalk cell to flip and become a new tip cell. In the first pattern, the tip cell required to be inhibited for the flip to occur is cell 4, and in the second arrangement, cell 5 (furthermore called the ‘correct cell’ inhibited). The stalk cell that flips is necessarily cell 5 and cell 4 respectively, as the others all still have a strong tip cell neighbour. Fig. 5(A) shows that the correct cell inhibited causes a flip in fate of the neighbouring stalk cell to a new tip, forming a second loop in the majority of cases; if the incorrect tip cell is inhibited, which could happen due to stochastic events, i.e. cell 2 or 7 respectively, then no second loop is observed. An arrangement such as S T S T S T S was found to never result in two loops; a flip of any tip cell to a stalk cell results in an equally viable arrangement, thus no stalk cell will flip.

Using VEGFR-2 levels as a marker for cell fate, it can be clearly seen from Fig. 5(B) that a cell's fate changes during the simulation. Once the first loop forms, the central cell indeed flips fate, triggering the second loop formation. Fig. 5(C) shows that when 10 times the normal VEGF level is used, the behaviour of the system is markedly different; a synchronous oscillation is observed. Interestingly under these pathological conditions, although new junctions form at a continuous rate, flipping of cell fates does not occur: oscillations are impervious to the changes in the network caused by anastomosis. The higher incidence of new junction springs in high VEGF, leads to sheet formation of multiple tip cells, rather than simple fusion of two non-adjacent tip cells. This is due to all cells attempting to become tip cells during each period of the oscillation and fusing together. See video S4 for a typical simulation in high VEGF. This emergent sheet-like sprouting morphology is interestingly consistent with observations of developing mouse retinas in conditions of excessive VEGF concentrations by intraocular injection [2].

### Junction boundary size can regulate Dll4-Notch tip cell selection in abnormally high VEGF

**Figure 6:**
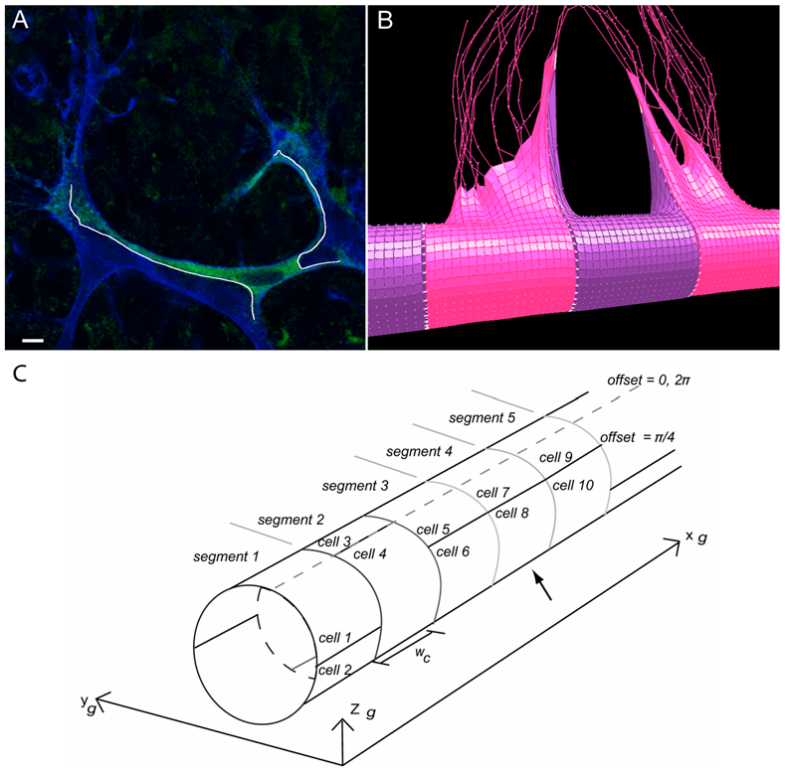
*In vivo* and *in silico* elongation of cell-cell junctions by migrating tip cells. (A) Confocal image of two tip cells (blue - isolectin B4 staining) in the mouse retina, stretching the stalk cell between them (green - Transgenic Notch Reporter signal eGFP), and significantly elongating the junction (highlighted in white). 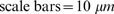 (B) This morphology, where a thin edge of the stalk cell lines the tip cell, is matched in simulation due to the low mesh spring constant. Tip cells - pink, stalk cells - purple, junction springs: white. (C) Diagram showing the vessel divided into segments to give two cells per cross section. A single offset parameter defines the position of the segments two cells. The offset runs from 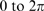, where zero divides them along the top of the vessel as indicated by the dotted line. Here the offsets are, in order of segments, 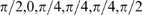. Having three equal offsets in a row leads the central cells (indicated by an arrow) to have only three neighbours whereas the cells in the outer segments will have five neighbours.

Mathematical models of lateral inhibition tend to naturally focus on regular, symmetric arrangements of cells to avoid any intrinsic or unintended bias [44],[45]. However, the junctions between real endothelial cells during sprouting are known to be highly contorted, irregular and indeed dynamic as cells stretch and move, as seen in Fig. 6(A,B), [3]. This irregularity could, we hypothesised, lead the unequal Dll4-Notch interactions to bias certain cells to become tips and thus stabilise a salt and pepper pattern faster. To investigate this, cells were initiated with irregular junctions and the stability of the system evaluated. Contrary to our initial simple hypothesis however, the emergent behaviour of the system exposed a far more important, subtle role for dynamic junction size in robustness of tip/stalk patterning.

The simulations were performed on a vessel comprising ten segment regions (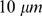 wide), but instead of segments representing one cell as before, they were divided into two cells. This facilitated unequal boundaries between neighbouring cells. Four distinct arrangements of cells were used: two ‘control’ arrangements, with regular, equal sized junctions and two irregular settings. These different arrangements were achieved by simply assigning a boundary offset parameter (in radians) for the position of the junction between each segment's two cells, see Fig. 6(C). The ‘Unequal Neighbours’ setting was expected to give the fastest stabilisation, due to the extra bias of different number of neighbour cells. As the cells were now half the size of those in previous simulations, the receptor and ligand levels per cell were also halved.

#### No Offset

Control: equal junction sizes. Three neighbours per cell (vertex only neighbours were discounted). Offset set to zero for all segments.

#### Equal Offset

Control: equal junction sizes. Five neighbours per cell. 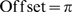 for even numbered segments, zero otherwise.

#### Unequal Offset

Unequal junction sizes. Five neighbours per cell. Offsets for even number segments 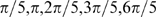, zero otherwise.

#### Unequal Neighbours

Unequal junction sizes. unequal neighbours, three, four or five per cell. Offsets for even number segments 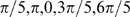, zero otherwise.

**Figure 7:**
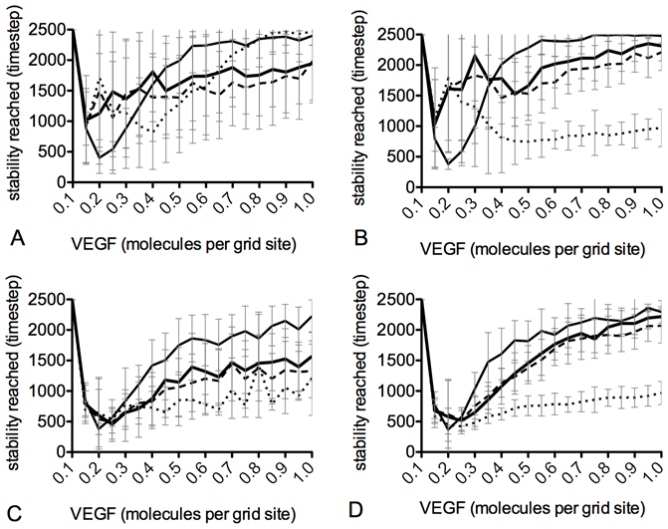
Graphs comparing selection stabilisation rates. Graphs showing the timestep when a salt and pepper pattern had been stable for at least 100 timesteps. Thin line - no offset, thick line - unequal neighbours, dotted line - equal offset, dashed line - unequal offset. (A) with migration, (B) no migration. Simulations were repeated but outlier runs which did not stabilise were removed. With migration (C) and without migration (D).

To avoid anastomosis events complicating the results with fate flipping, a linear gradient of VEGF, on a square astrocyte lattice, *without* point sources, was used. The VEGF concentration was varied between 0.1 and 1.0 molecule per grid site, in 0.1 steps, and results averaged over 50 runs of 2500 time steps at each VEGF setting. When a particular salt and pepper pattern had persisted for 100 time steps the timestep was recorded as the time stability was reached. The simulations were then all repeated with the migration of cells switched off, by placing a permanent adhesion at the base of filopodia, inhibiting cell body advance, and results compared to elucidate any effects of migration on junctions and stability. Fig. 7(A) shows that both unequal junction arrangements with migration fair the best in terms of quickly forming a stable pattern, particularly in simulations run at higher VEGF concentrations. However, there are surprisingly large error bars, particularly in lower VEGF levels, rendering conclusions unreliable. The occurrence of such variability in stabilisation rate was intruiging and unexpected. Thus a deeper evaluation of this variability was performed.

#### Investigation of stabilisation variability

The above variability in stabilisation score was found to be due to outlier runs which failed to stabilise. As they failed to stabilise they received a score of 2500 (the maximum number of time steps), which then skewed the standard deviation. The simulations were repeated but this time each run was classified as either ‘stabilised’ or ‘outlier’. Figs. 7(C) and (D) show the stability scores for the stabilised runs with outliers removed. Error bars, particularly in lower VEGF settings, are significantly reduced and the trends are clearer to distinguish. Surprisingly, the ‘Equal Offset’ control arrangement was consistently faster to stabilise than the others, with the other control arrangement , ‘No Offset’, the other regular arrangement, being the slowest. This suggested something other than regularity of junction size was behind the increased rate.

We hypothesised three reasons why a stable pattern could not be reached by outliers: 1) not enough VEGF is present so lateral inhibition cannot function; 2) too much VEGF causes an oscillation of cell fates; or 3) a stable salt and pepper pattern has occurred along most of the vessel except for two adjacent tip cells persisting. This third case could theoretically arise as Dll4 and Notch are equally distributed around each cells periphery, meaning that two cells which border along a small junction may not present enough Dll4/Notch to each other to establish a clear tip/stalk distinction. To investigate this, three criteria of outlier runs were assessed: 1) how long the pattern present at the final timestep had been stable for, see Supporting Information (text S1) for method; 2) the number of adjacent tip cells present and; 3) the average size of the junction between any adjacent tip cells selected.

**Figure 8:**
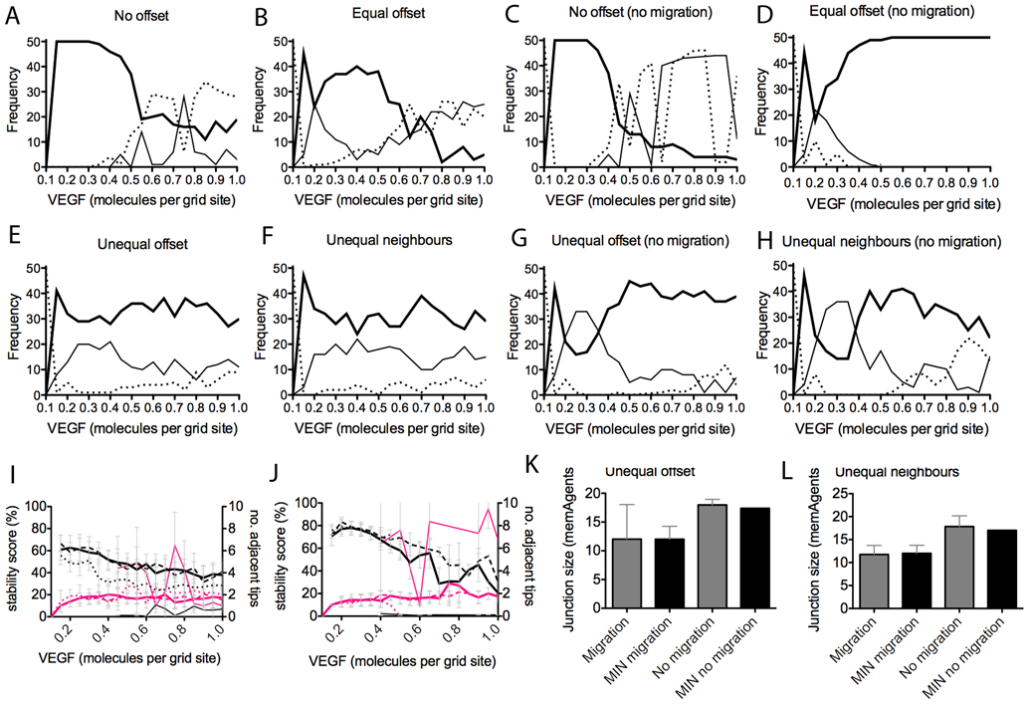
Frequency of runs which could not stabilise (outliers) for each junction arrangement setting. (A–H)Thick line - stabilised runs, thin line - outlier runs with at least two tip cells adjacent, dotted line - outlier runs with no adjacent tip cells. (I,J) Outliers tend to have actually stabilised (not oscillating) but with just one or two sets of two adjacent tip cells (pink line), thin line - no offset, thick line - unequal neighbours, dotted line - equal offset, dashed line - unequal offset. (I) with migration, (J) no migration. (K,L) Adjacent tip cells are shown to share the smallest junction possible in that cell arrangement. Actual smallest junction size possible (MIN) and the sizes of junctions between adjacent tip cells in outlier runs, observed for unequal offset and unequal neighbour arrangements respectively (averaged over 50 runs).

Outliers were then divided into two groups, those with or without adjacent tip cells. Fig. 8(A–H) shows the frequency of stabilised runs compared to these two outlier types. Clearly, the type of arrangement affects the likelihood of outliers. Both control arrangements, with equal junction sizes, produce more outlier runs as VEGF levels increase. The unequal arrangements, when migration is switched off, produce many outliers in low VEGF, but their ability to stabilise is surprisingly enhanced by increasing levels.

As summarised in Fig. 8(I) and (J), our hypothesis that outliers have three possible causes appears to hold. For both unequal arrangements, where adjacent tip cells are present, the pattern achieved has actually been stable for approximately half the run time. However, the stable pattern is not classical salt and pepper. One, or occasionally two pairs of adjacent tip cells are present. Fig. 8(K) and (L) show that the junctions between these adjacent tip cells are indeed the smallest junctions present in that arrangement. Rather than an incidence of adjacent tip cells, the ‘No Offset’ outliers, are clearly due to oscillations occurring; here the stability score is extremely low and the number of adjacent tip cells high.

#### Implications of junction size and normalisation by reduced migration

The above observations concerning outlier events point to an alternative hypothesis, that it is not uniformity/irregularity of junction size that is important for stabilisation rate, but simply, the actual size of the junctions. Indeed we can make the assertion that for a salt and pepper pattern to stabilise quickly, junction size should be inversely proportional to the amount of VEGF.

**Figure 9:**
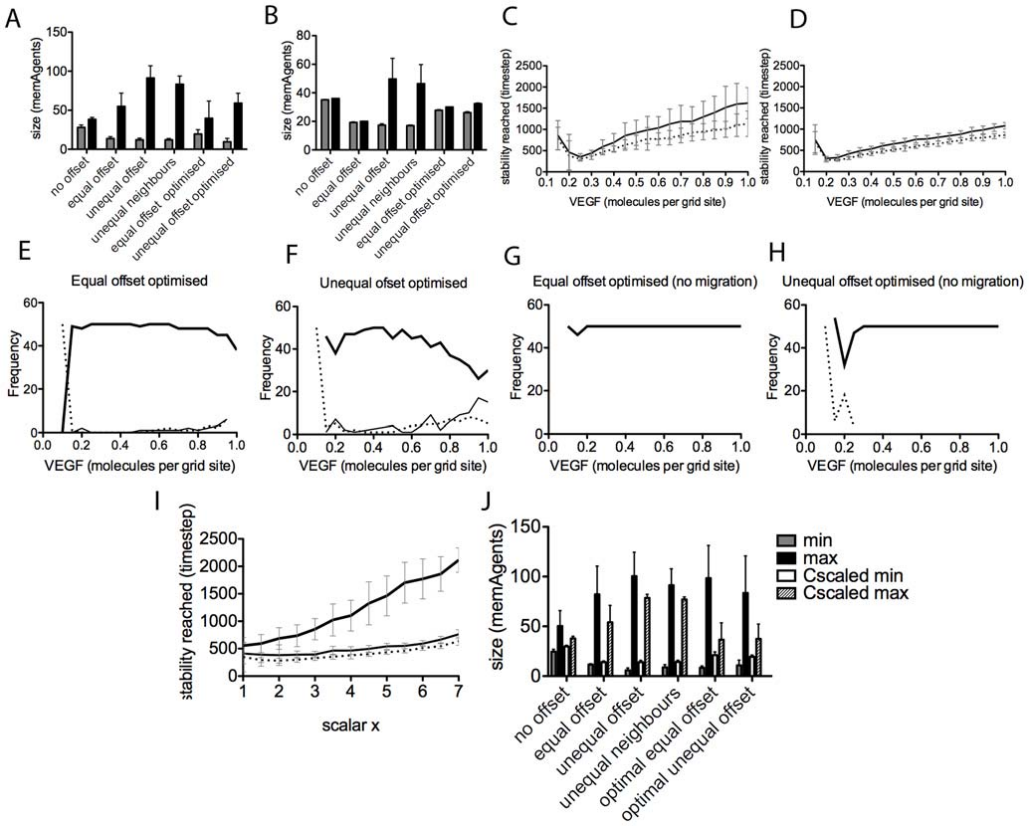
Graphs comparing junction size range and improved behaviour of optimised junction arrangements compared with previous arrangements. (A) minimum (grey) and maximum (black) junction sizes (measured by counting the number of memAgents along a junction, for adjacent cells if both are exposed to VEGF), averaged over 50 runs, no migration, (B) with migration. (C) Timestep at which stable runs reached the salt and pepper pattern with the optimised equal offset (dotted line) and optimized unequal offset (thin line) arrangements, with migration. (D) no migration. (E–H) frequency plots showing that the optimised arrangements have little or no outlier runs. Thick line - stable runs, thin line - outlier with adjacent tip cells, dotted line - outliers with no adjacent tip cells. (I) Graph showing that Reducing the C parameter, which scales the probability of being awarded an actin token upon receptor activation, by the same amount that VEGF has been increased by (scalar x) allows the two optimal junction arrangements to stabilise the salt and pepper pattern faster, even into very high VEGF (upto 7 times the normal setting of 0.25 molecules per grid site). Thin line - unequal optimal offset; dotted line - equal optimal offset. Scaling the C parameter, however, does not increase stabilisation rate of the no-offset arrangement as VEGF increases, as the difference is too small between minimum and maximum junction size in this arrangement. (J) the minimum and maximum junction sizes at t = 2500 when VEGF = 4 times normal setting and C is comparably scaled to one fourth its original value. With C-scaling the maximum is reduced and the minimum increased.

With a smaller junction size between two cells, they experience less Dll4 from each other (as Dll4 is equally distributed around the cells entire boundary of junctions). Therefore, in lower VEGF environments, two cells with a small junction may not be able to inhibit each other enough to differentiate. Equally, if the junction is large, in high VEGF the high Dll4 levels between the cells will form a prerequisite for oscillation, as seen in the ‘No Offset’ arrangement. Figs. 9(A) and (B) show that, even with migration (which clearly increases the range of junction sizes), the minimum junction sizes in this arrangement are greater than in all other arrangements; it rapidly descends into oscillatory behaviour. The arrangement with the most condensed junction size range is Equal Offset, see Fig. 9(A) and (B), and is indeed observed to stabilise the fastest with and without migration.

Thus, a small junction in high VEGF is advantageous, as the smaller proportion of Dll4 presented means oscillations are avoided. Interestingly, this suggests that a reduction in junction size alone is equivalent to a reduction in Dll4 levels, which is known to normalise angiogenesis in high VEGF environments [46]–[48]. Two optimised junction arrangements were engineered to test this. From Fig. 9(A) and (B) it was hypothesised that both maximum and minimum junction size for these arrangements should be just under thirty memAgents to achieve optimal results. In the first optimised setting, with equal junctions, all junctions were initially set to 27 memAgents long (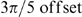). The second had unequal junctions ranging between 27–32 memAgents (using the following offsets per segment in the vessel: 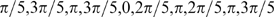). With migration this of course stretched to a wider range. The optimised settings show a stark improvement in the stabilisation rate (Fig. 9(C) and (D)) and reduction in adjacent incidents across VEGF levels (Fig. 9(E–H), showing this theory to be correct. However, with migration the widening means some outliers still occur.

It becomes clear that reducing migration will reduce junction stretching, facilitating stable selection into higher VEGF. Fig. 9(I) shows fast selection is indeed possible with migration reduced (parameter 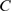 in the probabilistic equation determining activation of actin, see [31] or Supplementary text S1) by the same scalar that VEGF is increased by. 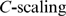 clearly makes a big improvement in both: 1) increasing the minimum junction size; and 2) reducing the maximum for all arrangements, Fig. 9(J).

One approach to reduce the actin-migratory response *in vivo* is reduce actin polymerisation using for example, SRF-inhibitors. SRF is a transcription factor which controls, amongst other things, expression of cytokeletal proteins [49]. SRF activity, in endothelial cells, is restricted to those in forming capillaries. SRF inactivation in normal development results in altered tip cell morphology but the same number of tip cells, hence Notch and Dll4 expression are unaffected. SRF negative tip cells are more ballooned (less stretched), with less filopodia and weaker junctions [50], suggesting that tip cell migration is indeed impaired. Moreover, inhibition of endothelial cell migration is currently considered as a potential mechanisms to inhibit tumour angiogenesis [51]. Our observations now suggest that such an approach may instead have novel implications for the use in vessel normalization therapy. The effects on normalising oscillatory selection via changes in junction size have so far not been investigated experimentally.

### Predicting a role for filopodia adhesions in tip cell migration and anastomosis: matching *in vivo* data

Here we show that the ‘veil advance’ mechanism for migration and navigation, based on filopodia adhesion signalling described in [33], yields realistic tip cell morphology and behaviour during migration and anastomosis. Figure 3 shows characteristic retinal tip cell morphology; filopodia can be very long and curved. Often tip cells display large numbers of filopodia without any evidence of migration of the main cell body (Fig. 3(F) green arrow). Fig. 3(E) white arrow, and (F) yellow arrow, show that where filopodia from different tip cells appear to have met, significantly more cell body has been pulled up along them. Figure 3(D) shows that switching off the filopodia-led veil advance mechanism yields unrealistic tip cell morphology. This is for two reasons, firstly the filopodia are entirely straight, having no mechanism for curvature without adhesions. Secondly, with adhesions veil advance can be delayed until the optimal direction for migration has been found; instead, without adhesions, cells instantly begin migrating in all directions, along every filopodia, with multiple environmental cues this would be highly inefficient *in vivo*. Without the veil advance mechanism, or a similar process, the morphology indicated by the green arrow in Fig. 3(F), with filopodia but no cell body advance, would not be possible.

Subsequently, time lapse *in vivo* movies were taken of developing intersomitic vasculature in the trunk region of zebrafish embryos (the retina cannot currently be live imaged) to validate the simulated veil advance mechanism (see video S1). Quantifications were made in the zebrafish and simulations concerning: a) the number of contacts between tip cell filopodia; b) how long they remained in contact and c) how long it took between 1) the first contact made between filopodia and fusion of the two tip cells, and 2) the final filopodia contact and fusion of the two tip cells. See Supporting Information (text S4, fig S6 and S7) for the live imaging method, specific parameter settings and the methods used in the simulations.

**Figure 10:**
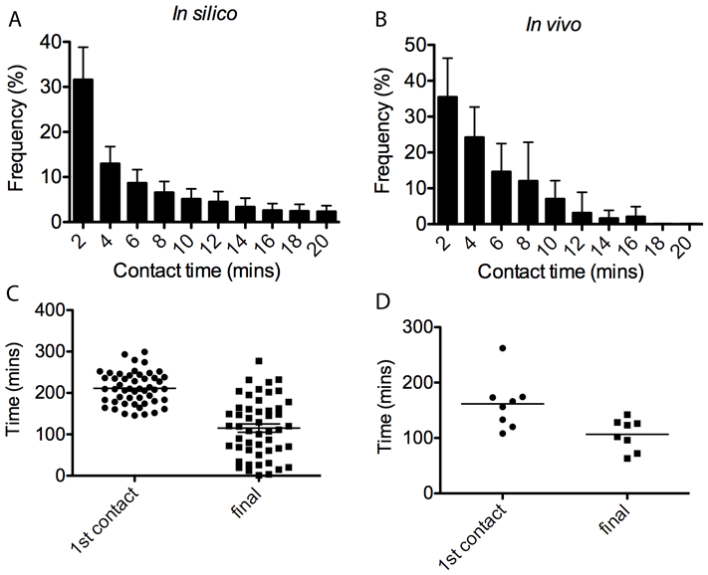
Graphs comparing *in vivo* and *in silico* filopodia contact data. (A) Frequency plot of simulation contact time distribution, averaged over 50 runs. (B) *in vivo* frequency, averaged over eight anastomosis events in live imaging of the developing zebrafish. (C) Scatter plot of the time from the first contact made until fusion and time from the last contact made until fusion, *in silico* and (D) *in vivo*.

The graphs in Fig. 10 show that a similar frequency distribution of contact lifetimes is obtained in simulation to *in vivo*, with a smoother curve in simulation due to averaging over fifty runs compared to eight anastomosis events *in vivo*. The *in silico* and *in vivo* contact time frequency were found to have no significant difference (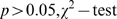). Fig. 10(C) and (D) show the time between the first filopodia contact and tip-tip fusion, and the time from the last filopodia contact and fusion are also similar in simulation to *in vivo*. It takes slightly longer to fuse from the first contact in simulation, however, the average time from final contact until fusion is almost exact.

#### Spring constant sensitivity analysis

The sensitivity of most model parameters were investigated in [31]. To test the sensitivity of the system to the new spring-based parameters, the following analysis was performed. The seven constants and ideal lengths, detailed in Table 1 were assigned a randomly chosen real value between 0.1 and 1. The system, with four cells as used in the previous section and described in text S4, was then evaluated after 2500 time steps. The resulting stage of development reached was classified as either: 1) failed (a salt and pepper pattern could not be achieved); 2) selected (a salt and pepper pattern has been reached and remained stable for 100 time steps, but no tip cell fusion occurs); 3) fused (a salt and pepper pattern was achieved and two tip cells fused); 4) flipped (a salt and pepper pattern was achieved and after two tip cells fused, one flipped fate to a stalk cell and remained stable for at least 50 time steps).

**Figure 11:**
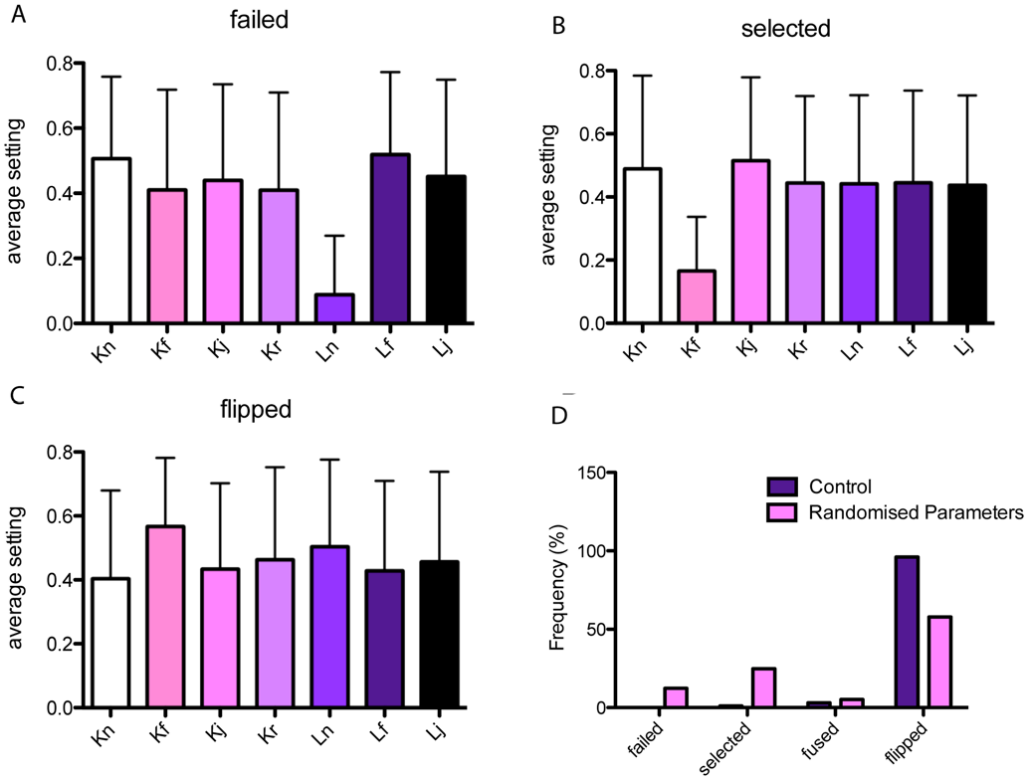
Spring constant sensitivity analysis results. The average setting for each spring parameter when the four stages of development are achieved by the final time step, see Table 1. (A) runs that failed to achieve a salt and pepper pattern noticeably have very low 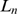 values. (B) runs that selected tip cells but did not fuse had very low 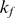 values, (C) runs where tip cells fused and one flipped fate show robustness to most parameter settings as long as the previously mentioned constants were set high. (D) Comparing the frequency of each possible outcome against runs with the parameter settings used in all other simulations, detailed in Table 1, which almost always show a flip in fate and never fail to select the salt and pepper pattern.

It is clear from Fig 11(A) that all runs which fail to achieve a salt and pepper pattern have a very low setting for 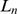, the ideal spring length in the general cell mesh. A low setting for this parameter generates excessive tension in the mesh and causes the cells to collapse inwards on themselves in an unrealistic manner, pulling them away from the VEGF gradient, thus no VEGFR-2 signalling occurs and the pattern is not generated. The ‘selected’ runs which failed to generate anastomosis clearly from Fig. 11(B) had a very low value for 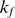 the parameter determining how quickly the veil is pulled up once stalk node adhesions have been released (filopodia spring constant). If this is too low then the cell bodies of the two cells will not reach each other to fuse before the final time step.

The majority of runs however did fuse and a flip in fate was observed, even across a wide range of parameter settings, Fig 11(C,D). Thus we can conclude that as long as both 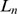 and 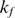 are both set reasonably high, the behaviour of the system is robust to changes in spring constant changes. However, although the system behaviour is functionally similar across different settings, not all will yield realistic morphological dynamics and rates for fusion. It is important that cells match morphological, confocal data as the spatial arrangement of cells affects the local levels of receptor activation, and the tension in the spring mesh will affect the speed of migration and fusion, which in turn determines the overall sprouting behaviour of the system. Hence the specific settings used both generated reliable functional behaviour but also matched the curvature, morphology and reasonable rates of migration and fusion based on close comparison with confocal data.

## Discussion

Here we have presented the significant extension of physical tension to our existing agent-based model, and considered the minimal, interlaced, pathways of ongoing lateral inhibition-driven tip cell selection, actin-driven migration and sprout fusion in normal and pathological angiogenesis. Through three separate investigations we arrived at a number of significant predictions. First, we reported emergent phenomena observed in simulations, showing anastomosis destabilises the established tip/stalk pattern and causes cell fates to flip. We also showed, in high VEGF, oscillations are robust to fusion events. Based on these simulation results the phrase ‘cell fate’ no longer seems appropriate for tip/stalk cells. Instead, given their reversible nature we suggest they be viewed as ‘phenotypes in flux’.

The second investigation focused on the effects of realistic, unequal cell-cell junction sizes on tip cell selection. This study gave surprising and intriguing results: if average junction size is inversely proportional to the VEGF level, then normal selection is possible, regardless of the VEGF level. Our initial hypothesis was that unequal junctions would simply increase selection rate due to the inherent bias. Instead, actual junction size, rather than relative size difference, was found to be a key, novel factor for fast and stable selection. Migration was shown to pull and stretch junctions, widening the range of sizes, which in turn feeds back to reduce stability of tip/stalk patterning in high VEGF. We showed that reducing migration, via the use of simulated actin polymerisation inhibitors, can reduce junction stretching and allow normal selection to occur in pathologically high VEGF. Lastly we quantitatively validated the novel filopodia-adhesion mechanism, seen only before in neuronal growth cones, against new *in vivo* live imaging data in zebrafish, establishing it as a plausible model deserving further investigation.

These results suggest migration is a key positive feedback loop involved in switching between normal and pathological angiogenesis. Figure 1(B) shows how the migration and selection protein pathways inter-relate. It is clear, just in terms of protein-pathways, that the positive feedback of migration will affect the functioning of the negative-feedback selection pathway; increased VEGFR-2 activity levels caused by migration will increase Dll4 expression, which can lead to abnormal oscillations. Positive feedback is known to make oscillations generated by negative feedback more robust in other biological systems, e.g. in the cell cycle, circadian rhythms and in tunable synthetic biological systems [52]–[54]. Interestingly it appears that the angiogenic sprouting system may exhibit more robustness in its pathological behaviour than in its normal functioning and may explain why it exhibits such sensitivity to VEGF levels, falling into abnormal development at just two times the normal amount [55].

Modularity, the localisation of functionally distinct processes to independent pathways, is known to improve the robustness of a system [56]; mutations or perturbations in one process are unable to disrupt those in a separate pathway. However, the sprouting pathway architecture, Fig. 1(B) clearly exhibits a lack of modularity; VEGFR-2 is central to migration and selection. Disruptions in VEGFR-2 activation will cause both processes to be affected, which in turn affects fusion events and the network integrity.

It has been argued that biological systems, in particular morphogenesis, are intrinsically robust [56]–[58]. Thus, it is interesting to uncover a case where the system shows sensitivity under normal conditions and may instead fall into robust pathological behaviour. However, it has also been argued that robustness may be a by-product of morphogenesis evolution, rather than directly selected for [59]; as such its omission in a particular developmental process is not unlikely, if a non-robust mechanism had other selective advantages. Indeed we have shown that the sensitivity of selection to fusion events, disrupting cell fates, has the advantage that new tip cells are rapidly selected, allowing the recursive generation of sprout loops, without requiring a long wait for cell division to provide new tips. Insights into such trade-offs between robustness and performance, and why pathological systems fail, has been highlighted for its therapeutic value [56]. Here it has enabled us to arrive at a new insight: that therapeutic intervention of the migration pathways in endothelial cells could aid normalisation of angiogenesis in diseases characterised by high VEGF levels. Our computer model, with its unique blend of spatial cell migratory morphological plasticity and local signal interpretation capacity, is central to making these observations, which could not have been achieved with any other model currently available.

Nevertheless, as with all computer models, development is an ongoing and cyclical process, predictions lead to more data which can then be incorporated to further improve the realism and predictive power of the model. Indeed the model is also designed for both extension and translation to other biological domains involving migration and social interaction of cells. Many more features, pathways and mechanisms are intended to be included in future development of the model. Also new experiments are being devised to test predictions and novel questions raised by the model. For example, we assume that Dll4 and Notch are evenly distributed around a cell's junctions, leading to the importance of junction size in pattern selection. In future work we intend to investigate whether cells can localise or cluster these proteins. However, as sprouting is disrupted in high VEGF it follows that even distribution is more likely.

Cell growth and division were excluded in this study to maintain focus on the early, necessary and sufficient stages of sprouting: migration, fusion and lateral inhibition. Cell division is not necessary for initial sprouting and fusion in early plexus formation [60]. Moreover, stalk cell division was not expected to cause disruption to selection. If the stalk cell dividing already neighbours another stalk cell then the row of three produced will cause the central one to default to a tip cell without destabilising the other tip cells nearby. However, division may well affect local junction arrangements as the new cells shuffle into position, thus impacting on the robustness of tip/stalk patterning.

The spring agent model described here, is ideally suited for extension to include growth and division. New rows, or columns, of nodes and springs can be inserted to mimic cell growth; the mesh can be divided into two for division. Further more, we aim to investigate the system in terms of more dynamic and complex environments. Currently to avoid unintended bias and keep the model simple the gradients of VEGF have been kept static. However, now the model has been fully validated in this simple setup, multiple competing, diffusing signals in the environment can be explored. Indeed utilising this systems biology approach, with a complex dynamic environment, may prove fundamental to understanding the careful balance needed to normalise angiogenesis across the spectrum of complex, pathological conditions.

## Supporting Information

Figure S1 Curvature of sprouts in vivo caused by cell tension, not strict adherence to astrocyte prepattern. (A)–(C) Screenshots from the movie supplied in video S5 of developing mouse retina vasculature. The endothelial cells (pink; isolectin B staining), at certain points overlap the astrocytes (blue; PDGFR staining), indicated with arrows, showing the curvature of the blood vessels is due to cell tension rather than strict adherence to the astrocytes. (D)–(E) Screens shots of the model zoomed in to show the endothelial cells in the model (black) also overlap the astrocytes (blue) due to tension in the mesh rather than strict adherence to the astrocyte prepattern, as exhibited in the retina. (F)–(G) Confocal images of the retina with astrocytes in blue) and endothelial cells (pink). (F) Only endothelial cells shown. In (G) it is clear that regions with the endothelial cells have much thicker astrocytes, which closely match the vascular morphology, whereas the top area, which has no endothelial cells, has much thinner astrocytes. This variation in astrocyte thickness is caused by the astrocytes remodelling and adapting their shape to fit with the endothelial cells lying on top of them.
(1.49 MB TIF)

Figure S2 Spring positional labelling. The following notation is used for the four connecting nodes to any given node P, Nrh,Nlh,Nrv,Nlv, where subscripts indicate positions, right horizontal, left horizontal, right vertical, left vertical. This notation clarifies procedures associated with mesh manipulation algorithms such as the surface agent coverage and cell growth functionalities. (B) For surface agent coverage, the upper and lower triangles from a node P are defined by the above spring positions.
(0.41 MB EPS)

Figure S3 The voxelisation process for surface agent coverage. A surface agent is created at every grid site position that each mesh triangle intersects. For each grid site near the triangle the following two tests are performed: triangle-line/cube-plane intersection and triangle-plane/cube-diagonal intersection.
(0.43 MB EPS)

Figure S4 Spring agent coverage. The grid sites which the springs pass through have a non-node agent created in them. Each timestep this coverage process is repeated to maintain an upto date continuous coverage of the membrane.
(0.45 MB EPS)

Figure S5 The voxelised mesh. A two-dimensional representation of a single cell. Three screen shots through one simulation of the model showing surface and spring agent coverage. Node agents black, surface agents dark grey, spring agents light grey.
(0.08 MB TIF)

Figure S6 Frames from time lapse microscopy of the developing zebrafish embryo. Frames taken every 2 minutes. Initially filopodia appear to be inhibiting veil advance. Arrow points to veil advance occurring on a tip cell (left), once initial contact between its filopodia ad another tip cell's (right) has been made.
(8.27 MB TIF)

Figure S7 In vivo time-lapse confocal images of intersegmental vessel during anastomosis in zebrafish embryo. Arrow in (B) indicates contact site. Two minutes elapsed between images. Images are 2D projections of 55 optical depth sections taken with a spinning disk confocal system, 60×.
(0.92 MB TIF)

Text S1 The memAgent model.
(0.04 MB PDF)

Text S2 Supporting evidence that cell membrane curvature is driven by tension in the actin cortex not astrocyte pre-pattern.
(0.02 MB PDF)

Text S3 Voxelisation method.
(0.02 MB PDF)

Text S4 Live imaging and simulation method specifics for quantification of the contact-veil advance filopodia mechanism.
(0.03 MB PDF)

Video S1 Time-lapse confocal microscopy of intersegmental tip cells during anastomosis in the zebrafish embryo. 2D projections of 55 optical depth sections taken with a spinning disk confocal system, 60×.
(7.53 MB MOV)

Video S2 Simulation showing fusing tip cell becoming inhibited. Movie of simulation on A shape retina section astrocytes, with point source at the top of the A frame, showing two tip cells being selected, migrating and fusing. Upon fusion both tip cells begin to inhibit each other, briefly disrupting the stable selection pattern. Eventually a new, stable state, is reached with one tip cell flipping to the alternate cell fate, a stalk cell.
(6.59 MB MP4)

Video S3 Simulation showing stalk cell flipping fate and facilitating a second loop. Simulation of a 7 cell vessel, showing flipping of tip, and neighbouring stalk cell fates, upon anastomosis.
(6.34 MB MP4)

Video S4 Simulation showing oscillation in high VEGF. Simulation, for a 7 cell vessel, within a high VEGF (10×) environment showing synchronous oscillation of all cells between all tip and all stalk cells. During the all tip phase, cells migrate and fuse in a realistic abnormal sheet-like morphology.
(2.62 MB MP4)

Video S5 Movie of confocal image showing membrane curvature. Movie fading the pink channel (isolectin B staining) to show the endothelial cells at certain points overlap the astrocytes (blue; PDGFRα staining) rather than strictly following their curvature.
(5.68 MB MOV)

## References

[1] Risau W (1997). Mechanisms of angiogenesis.. Nature.

[2] Gerhardt H, Golding M, Fruttiger M, Ruhrberg C, Lundkvist A (2003). VEGF guides angiogenic sprouting utilizing endothelial tip cell filopodia.. Journal of Cell Biology.

[3] Blum Y, Belting HG, Ellertsdottir E, Herwig L, Lüders F (2008). Complex cell rearrangements during intersegmental vessel sprouting and vessel fusion in the zebrafish embryo.. Developmental Biology.

[4] Djonov V, Makanya AN, Clauss M, Breier G (2005). New insights into intussusceptive angiogenesis.. Mechanisms of angiogenesis.

[5] Koganehira Y, Takeoka M, Ehara T, Sasaki K, Murata H (2003). Reduced expression of actin-binding proteins, h-caldesmon and calponin h1, in the vascular smooth muscle inside melanoma lesions: an adverse prognostic factor for malignant melanoma.. Br J Dermatol.

[6] Jain R (2005). Normalization of tumour vasculature: an emerging concept in antiangiogenic therapy.. Science.

[7] Tyson J (2007). Bringing cartoons to life.. Nature.

[8] Lewis J (2008). From signals to patterns: space, time and mathematics in developmental biology.. Science.

[9] Anderson ARA, Chaplain MAJ (1997). A mathematical model for capillary network formation in the absence of endothelial cell proliferation.. App Math Letters.

[10] Levine HA, Sleeman BD, Nilson-Hamilton M (2001). Mathematical modelling of the onset of capillary formation initiating angiogenesis.. Journal of Mathematical Biology.

[11] McDougall SR, Anderson ARA, Chaplain MAJ (2006). Mathematical modelling of dynamic adaptive tumour-induced angiogenesis: Clinical implications and therapeutic targeting strategies.. Journal of Theoretical Biology.

[12] Pierce SM, Skalak TC, Papin JA (2006). Multiscale biosystems integration: coupling intracellular network analysis with tissue-patterning simulations.. IBM J Res & Dev.

[13] Mac Gabhann F, Popel AS (2006). Targeting neuropilin-1 to inhibit vegf signalling in cancer: comparison of therapeutic approaches.. PLOS Computational Biology.

[14] Owen M, Alarcon T, Maini PK, Byrne H (2009). Angiogenesis and vascular remodelling in normal and cancerous tissues.. J Math Biol.

[15] Bauer AL, Jackson TL, Jiang Y (2007). A cell-based model exhibiting branching and anastomosis during tumour-induced angiogenesis.. Biophys J.

[16] Meinhardt H (1976). Morphogenesis of lines and nets.. Differentiation.

[17] Addison-Smith B, McElwain DLS, Maini PK (2008). A simple mechanistic model of sprout spacing in tumour-associated angiogenesis.. J Theor Biol.

[18] Merks RMH, Perry ED, Shirinifard A, Glazier JA (2008). Contact-inhibited chemotaxis in de novo and sprouting blood-vessel growth.. PLoS Computational Biology.

[19] Hellström M, Phng L-K, Hofmann JJ, Wallgard E, Coultas L (2007). Dll4/notch1 signalling is required for selection of single endothelial tip cells during angiogenic sprouting.. Nature.

[20] Qutub AA, Popel AS (2009). Elongation, proliferation and migration differentiate endothelial cell phenotypes and determine capillary sprouting.. BMC sys biol.

[21] Bottino D, Mogilner A, Roberts T, Stewart M, Oster G (2002). How nematode sperm crawl.. Journal of Cell Science.

[22] Mi Q, Swigon D, Riviére B, Cetin S, Vodovotz Y (2007). One-dimensional elastic continuum model of enterocyte layer migration.. Biophysical Journal.

[23] Meineke FA, Potten CS, Loeffler M (2001). Cell migration and organization in the intestinal crypt using a lattice-free model.. Cell Prolif.

[24] Lamalice L, Houle F, Jourdan G, Huot J (2004). Phosphorylation of tyrosine 1214 on vegfr2 is required for vegf-induced activation of cdc42 upstream of sapk2/p38.. Oncogene.

[25] Rousseau S, Houle F, Landry J, Huot J (1997). p38 map kinase activation by vascular endothelial growth factor mediates actin reorganization and cell migration in human endothelial cells.. Oncogene.

[26] Graupera M, Guillermet-Guibert J, Foukas LC, Phng L-K, Cain RJ (2008). Angiogenesis selectively requires the p110 *α* isofrom of pi3k to control endothelial cell migration.. Nature.

[27] Liu Z-J, Shirakawa T, Li Y, Soma A, Oka M (2003). Regulation of *Notch1* and *Dll4* by vascular endothelial growth factor in arterial endothelial cells: implications for modulating arteriogenesis and angiogenesis.. Molecular and Cellular Biology.

[28] Williams CK, Li J-L, Murga M, Harris AL, Tosato G (2006). Up-regulation of the notch ligand delta-like 4 inhibits VEGF-induced endothelial cell function.. Blood.

[29] Sainson R, Aoto J, Nakatsu MN, Holderfield M, Conn E (2005). Cell-autonomous notch signalling regulates endothelial cell branching and proliferation during vascular tubulogenesis.. FASEB J.

[30] Sainson RCA, Harris AL (2006). Hypoxia-regulated differentiation: let's step it up a notch.. TRENDS in molecular medicine.

[31] Bentley K, Gerhardt H, Bates PA (2008). Agent-based simulation of notch mediated tip cell selection in angiogenic sprout initialisation.. Journal of Theoretical Biology.

[32] Stricker J, Cookson S, Bennett MR, Mather WH, Tsimring LS (2008). A fast, robust and tunable synthetic gene oscillator.. Nature.

[33] Steketee MB, Tosney KW (2002). Three functionally distinct adhesions in filopodia: Shaft adhesions control lamellar extension.. The Journal of Neuroscience.

[34] Heidermann SR, Wirtz D (2004). Towards a regional approach to cell mechanics.. TRENDS in Cell Biology.

[35] Freitas R (1999). Nanomedicine, Volume I: Basic Capabilities.

[36] Mac Gabhann F, Ji JW, Popel AS (2006). Computational model of VEGF spatial distribution in muscle and pro-angiogenic cell therapy.. PLoS Computational Biology.

[37] Alberts B, Bray D, Lewis J, Raff M, Roberts K (1994). Molecular Biology of The Cell.

[38] Mogilner A, Rubinstein B (2005). The physics of filopodia protrusion.. Biophys J.

[39] Svitkina TM, Bulanova EA, Chaga OY, Vignjevic DM, Kojima S-IBorisy GG (2003). Mechanism of filopodia initiation by reorganization of a dendritic network.. J Cell Biol.

[40] Kress H, Stelzer EHK, Holzer D, Buss F, Griffiths G (2007). Filopodia act as phogocytic tentacles and pull with discrete steps and a load-dependent velocity.. PNAS.

[41] Webb DJ, Parsons T, Horwitz AF (2002). Adhesion assembly, disassembly and turnover in migrating cells - over and over again.. Nature Cell Biology.

[42] Gariano RF, Gardner TW (2004). Retinal angiogenesis in development and disease.. Nature.

[43] Stockman C, Doedens A, Weidemann A, Zhang N, Takeda N (2009). Deletion of vascular endothelial growth factor in myeloid cells accelerates tumorigenesis.. Nature.

[44] Collier JR, Monk NAM, Maini PK, Lewis J (1996). Pattern formation by lateral inhibition with feedback: a mathematical model of delta-notch intercellular signalling.. J Theor Biol.

[45] Di Ventura B, Lemerle C, Michalodimitrakis K, Serrano L (2006). From *in vivo* to *in silico* biology and back.. Nature.

[46] Sainson RCA, Harris AL (2007). Anti-dll4 therapy: can we block tumour growth by increasing angiogenesis?. TRENDS in molecular medicine.

[47] Ridgway J, Zhang G, Wu Y, Stawicki S, Liang W-C (2006). Inhibition of dll4 signalling inhibits tumour growth by deregulating angiogenesis.. Nature.

[48] Noguera-Troise I, Daly C, Papadopoulos NJ, Coetzee S, Boland P (2006). Blockade of dll4 inhibits tumour growth by promoting non-productive angiogenesis.. Nature.

[49] Treisman R (1986). Identification of a protein-binding site that mediates transcriptional response of the c-fos gene to serum factors.. Cell.

[50] Franco CA, Mericskay M, Parlakian A, Gary-Bobo G, Gao-Li J (2008). Serum response factor is required for sprouting angiogenesis and vascular integrity.. Dev Cell.

[51] Chai J, Jones MK, Tarnawski AS (2004). Serum response factor is a crticail requirement for vegf signalling in endothelial cells and vegf-induced angiogeesis.. FASEB J.

[52] Gore J, van Oudenaarden A (2009). The yin and yang of nature.. Nature.

[53] Brandman O, Meyer T (2008). Feedback loops shape cellular signals in space and time.. Science.

[54] Tigges M, Marquez-Lago TT, Stelling J, Fussenegger M (2009). A tunable synthetic mammalian oscillator.. Nature.

[55] Carmeliet P, Ferreira V, Breier G, Pollefeyt S, Kieckens L (1996). Abnormal blood vessel development and lethality in embryos lacking a single VEGF allele.. Nature.

[56] Kitano H (2004). Space as the final frontier in stochastic simulations of biological systems.. Nat Rev Genet.

[57] Goodwin BC, Kauffman S, Murray JD (1993). Is morphogenesis an intrinsically robust process?. J Theor Biol.

[58] Wagner A (2005). Robustness, evolvability and neutrality.. FEBS Letters.

[59] Basanta D, Miodownik M, Baum B (2008). The evolution of robust development and homeostasis in artificial organisms.. PLoS Comp Biol.

[60] Ausprunk DH, Folkman J (1977). Migration and proliferation of endothelial cells in preformed and newly formed blood vessels during tumor angiogenesis.. Microvasc Res.

[61] Wang D, Lehman RE, Donner DB, Malti MR, Warren RS (2002). Expression and endocytosis of VEGF and its receptors in colonic vascular endothelial cells.. Am J Phsyiol Gastrointest Liver Physiol.

[62] Leslie JD, Ariza-McNaughton L, Bermange AL, McAdow R, Johnson SJ (2007). Endothelial signalling by the notch ligand delta-like 4 restricts angiogenesis.. Development.

