# Supplementary figures and images for: Tipping the Balance: Robustness of Tip Cell Selection, Migration and Fusion in Angiogenesis
Source: PLoS Comput Biol. 2009 Oct 30;5(10):e1000549. doi: 10.1371/journal.pcbi.1000549 (PMC2762315; doi:10.1371/journal.pcbi.1000549)

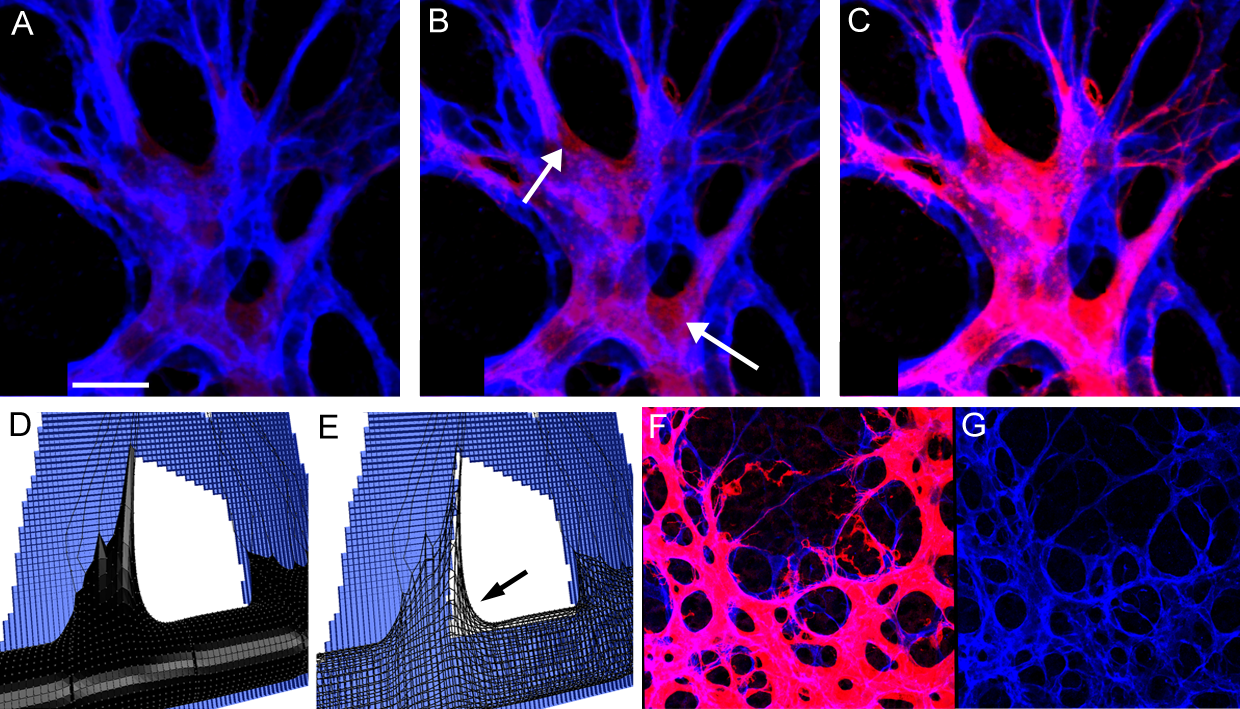

Supplement: Figure S1 — Curvature of sprouts in vivo caused by cell tension, not strict adherence to astrocyte prepattern. (A)–(C) Screenshots from the movie supplied in video S5 of developing mouse retina vasculature. The endothelial cells (pink; isolectin B staining), at certain points overlap the astrocytes (blue; PDGFR staining), indicated with arrows, showing the curvature of the blood vessels is due to cell tension rather than strict adherence to the astrocytes. (D)–(E) Screens shots of the model zoomed in to show the endothelial cells in the model (black) also overlap the astrocytes (blue) due to tension in the mesh rather than strict adherence to the astrocyte prepattern, as exhibited in the retina. (F)–(G) Confocal images of the retina with astrocytes in blue) and endothelial cells (pink). (F) Only endothelial cells shown. In (G) it is clear that regions with the endothelial cells have much thicker astrocytes, which closely match the vascular morphology, whereas the top area, which has no endothelial cells, has much thinner astrocytes. This variation in astrocyte thickness is caused by the astrocytes remodelling and adapting their shape to fit with the endothelial cells lying on top of them. (1.49 MB TIF) [file pcbi.1000549.s001.tif]

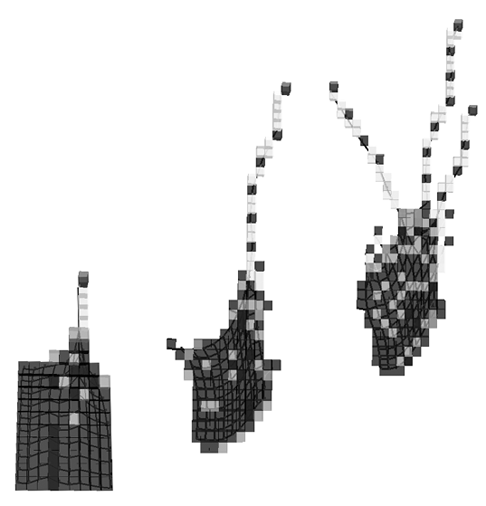

Supplement: Figure S5 — The voxelised mesh. A two-dimensional representation of a single cell. Three screen shots through one simulation of the model showing surface and spring agent coverage. Node agents black, surface agents dark grey, spring agents light grey. (0.08 MB TIF) [file pcbi.1000549.s005.tif]

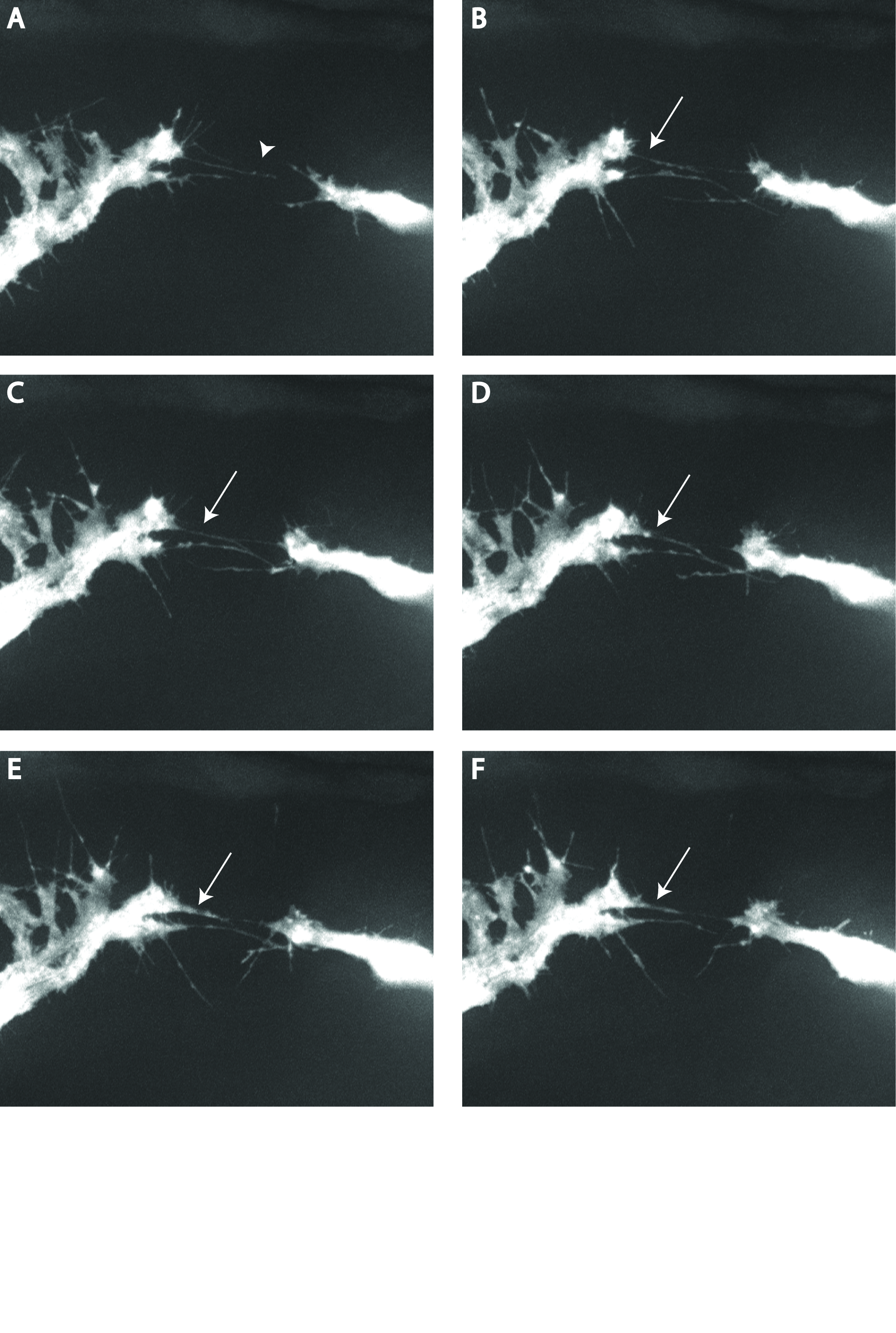

Supplement: Figure S6 — Frames from time lapse microscopy of the developing zebrafish embryo. Frames taken every 2 minutes. Initially filopodia appear to be inhibiting veil advance. Arrow points to veil advance occurring on a tip cell (left), once initial contact between its filopodia ad another tip cell's (right) has been made. (8.27 MB TIF) [file pcbi.1000549.s006.tif]

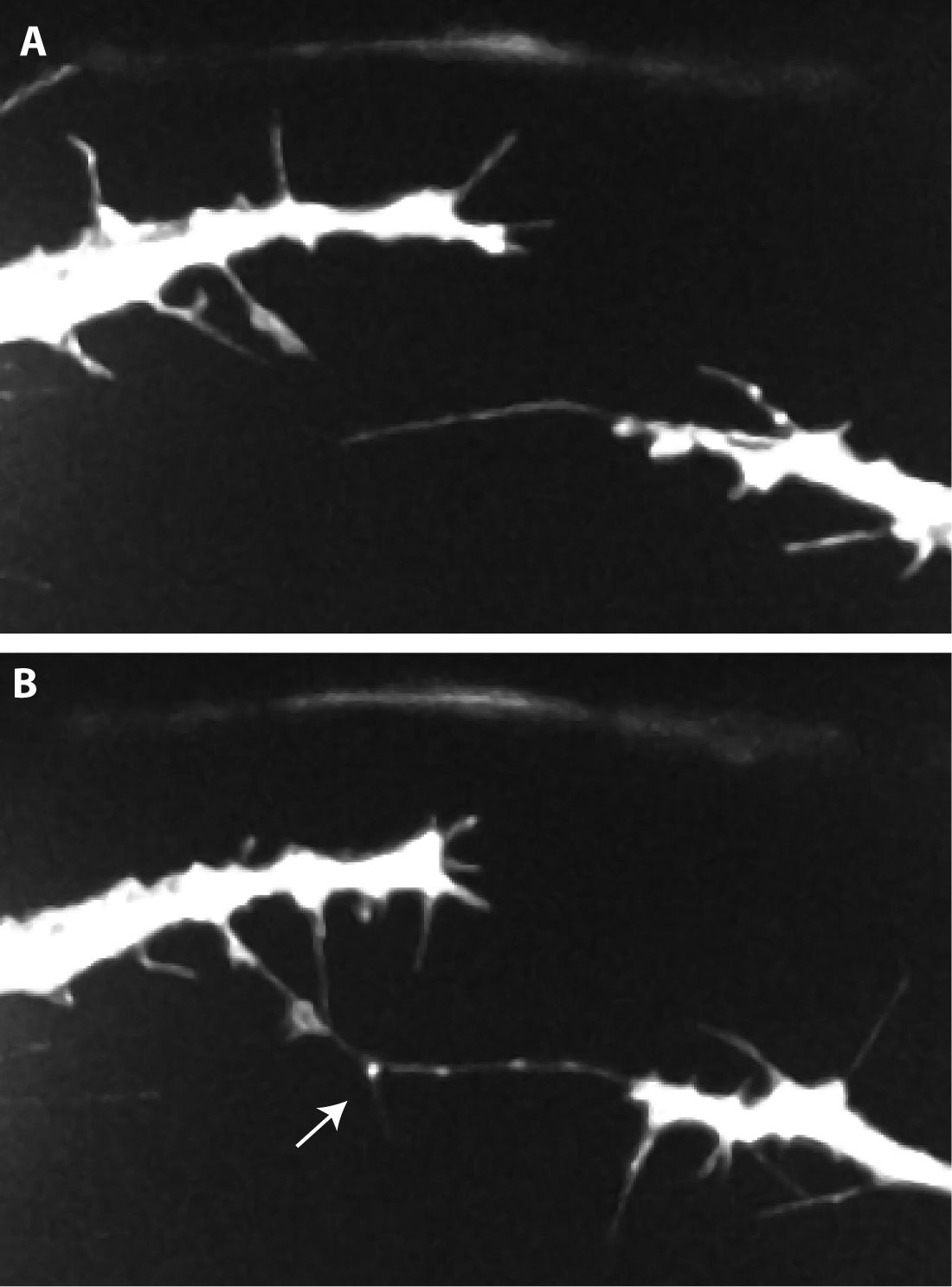

Supplement: Figure S7 — In vivo time-lapse confocal images of intersegmental vessel during anastomosis in zebrafish embryo. Arrow in (B) indicates contact site. Two minutes elapsed between images. Images are 2D projections of 55 optical depth sections taken with a spinning disk confocal system, 60×. (0.92 MB TIF) [file pcbi.1000549.s007.tif]
