# Supplementary material for: Tipping the Balance: Robustness of Tip Cell Selection, Migration and Fusion in Angiogenesis
Source: PLoS Comput Biol. 2009 Oct 30;5(10):e1000549. doi: 10.1371/journal.pcbi.1000549 (PMC2762315; doi:10.1371/journal.pcbi.1000549)
Supplement: Text S1 — The memAgent model. (0.04 MB PDF) [file pcbi.1000549.s008.pdf]

The memAgent model

Here we give a brief overview of the main features of the memAgent model, as described in [1], upon which the current model was built. Minor modifications to the original model, to fit the new, are outlined below; major additions to the original model have been described in the main body of the text.

MemAgents hold unique levels of receptors and ligands, determined at each timestep by the cell's genetic regulatory network, described below. The level of active receptor is updated by an assessment protocol which assesses the level of ligands for the receptor in the local neighbourhood. Given receptor activation, each memAgent accrues actin tokens which can then be used or passed to neighbours.

Each memAgent has a unique level of VEGFR2, Notch and Dll4, denoted by 'V', 'N' and 'D' respectively. Each cell agent know the total levels of these across all its memAgents. A full discussion, and justification based on experimental data in the literature, of receptor quanities, VEGF concentration and activation levels can be found in [1]. Here we use the same values, but scaled as only the ablumenal membrane surface is considered. In the memAgent model used in [1], 69% of the receptors were located on the ablumenal surface, thus V_{max} was set to 69% of the previous model setting, see Table 1. Intialising the mesh in a cylindrical form generates more memAgents than previously, so inorder to represent the actual size of the exposed surface correctly, only memAgents adjacent to an 'environment' grid site, sharing a grid site face (Von Neumann neighbourhood) were allowed to activate receptors, as was the case in the previous model (as only Von Neumann neighbourhood memAgents were initialised). The other memAgents are essentially in reserve and become exposed as the membrane surface changes due to migration. The above is consistent with the expected number of cell surface receptors and approximate cell surface area (750 memAgents).

To execute VEGFR-2 receptor activation, each memAgent assesses its local grid site neighbours n and sums the number of nearby $VEGF$ molecules to activate its VEGFR-2 receptors $V \rightarrow V'$. The number of active receptors V' is given by

$$V'_m = \begin{cases} V_{calc} & \text{if } V_{calc} < V \\ V_m & \text{otherwise} \end{cases}$$

where

$$V_{calc} = V_m/V_{m_{max}}(\sum_{n=1}^{n=26} VEGF_n/V_{sink})$$

where V_{sink} is a constant describing the proportion of available ligands that would be diverted away by binding to the sink receptor VEGFR-1, and $V_{m_{max}}$ is the maximum possible concentration for that receptor in a single memAgent, given the current number of memAgents in the cell (M_{tot}), so that

$$V_{m_{max}} = V_{max}/M_{tot}.$$

The activation of VEGFR-2 receptors is assumed to increase the local concentration of active actin for polymerisation, thus the stochastic function

$$P(actin) = CV'_m/V_{m_{max}} \ ,$$

is used to determine how much actin is available in that memAgents locality, in the form of abstracted actin 'tokens' awarded to that memAgent. Where C is a constant whose value controls the strength of the filopodia response. As this could unnecessarily result in a probability over 1, given a sufficently high value for C, we cap the probability such that if it is over 1 it is reset to 0.99; there is always a slight chance of not being awarded actin. Actin can then be used, if the memAgent is in the correct state, to extend filopodia. Previously a limit was set such that filopodia must be at least two microns apart,

however as the cells are now responding to VEGF in a more condensed region, along astroctye strutts, not all around the cell as in [1], this restriction was removed.

If the memAgent being updated is located at the cell-cell junction, it removes Dll4 from any neighbouring memAgents belonging to the other cell, in turn, up to the sum of its Notch level (N_m). If less Dll4 exists than a particular cell's available amount of Notch, then N'_m is set to the total Dll4 taken by that cell. If the amount of Dll4 in the neighbours exceeds N_m then $N'_m = N_m$.

The minimal timestep in the model represents approximately 15 seconds. This is based on the time it takes to extend a section of filopodia as long as one grid site, based on zebrafish data [1].

After all memAgents have updated, each cell updates its total active receptor levels V'_c and N'_c by summing the new memAgent values; these levels are able to affect future protein expression via a genetic regulatory network. Computational stacks represent the time it takes for transcription and translation events; an active receptor would not have an instant effect on gene expression. The stacks act as a delay between activation and regulatory effect. The current total values are added to the top of the delay stacks (initially set to zero height). If the V'_c stack has a height equal to delay D1, the bottom (oldest) value is considered effective and is removed and used to up-regulate Dll4. Similarly, N'_c values are entered onto a stack of height D2 and the bottom value, N''_c, is considered effective and used to reduce VEGFR-2 levels.

The delays in the model were set such that their sum was approximately fifteen minutes, as the period of oscillation for a similar Notch-delta inhibition system was found to be thirty minutes and, moreover, was shown to be equal to twice the sum of the delays involved [2]. D1 and D2 were set to 7 minutes each (28 timesteps). Each timestep, the following equations are used to determine the cell's new D_c and V_c levels (at timestep t):

$$D_{c_t} = D_{c_{t-1}} + (V''_c \times \delta) \quad (1)$$
$$V_{c_t} = V_{max} - (N''_c \times \sigma) \quad (2)$$

Once the new cell wide levels of receptors and ligands have been calculated, they are divided equally among the memAgents (Notch and Dll4 are only located in memAgents at a junction). This equal redistribution of proteins across the membrane, represents the average diffusion of proteins on the cell surface and follows the fluid mosaic model of biological membrane [3].

To evaluate the stability of a cell's fate, a binary score was given each timestep and for each cell. It would score 1 if $V_{c_t} = V_{c_{t-1}} \pm \rho$, and zero otherwise, where ρ was a significance value. At the final timestep the number of consecutive positive scores, working backwards, was averaged over each cell agent and given as a percentage of the overal run time.

References

[1] Bentley K, Gerhardt H, Bates PA (2008) Agent-based simulation of notch mediated tip cell selection in angiogenic sprout initialisation. Journal of Theoretical Biology 250: 25-36.

[2] Guidicelli F, Lewis J (2004) The vertebrate segmentation clock. Current Opinion in Genetics & Development 14: 407-414.

[3] Alberts B, Bray D, Lewis J, Raff M, Roberts K, et al. (1994) Molecular Biology of The Cell. Garland Publishing, 3rd edition.
