# Supplementary material for: Tipping the Balance: Robustness of Tip Cell Selection, Migration and Fusion in Angiogenesis
Source: PLoS Comput Biol. 2009 Oct 30;5(10):e1000549. doi: 10.1371/journal.pcbi.1000549 (PMC2762315; doi:10.1371/journal.pcbi.1000549)
Supplement: Text S2 — Supporting evidence that cell membrane curvature is driven by tension in the actin cortex not astrocyte pre-pattern. (0.02 MB PDF) [file pcbi.1000549.s009.pdf]

Cell membrane curvature is driven by tension not astrocyte pre-pattern

The fundamental premise of our spring-based model is that actin led protrusions combined with cell membrane tension provided by the actin cortex, and adherence to neighbour cells, causes the characteristic long stretched, curved morphology and migratory behaviour of tip cells. In order to confirm this we needed to rule out the null hypothesis that the curved thin morphology of developing sprouts is not tension driven but dictated by the astrocyte pre-pattern. If the null hypothesis were true then all curvature of the endothelial cells would be exactly the same as the astrocytes beneath them. However, as can be seen in Fig. S1, the endothelial cells overlap the curves of the astrocytes by some margin, see video S5 for a movie of this. Therefore, the basis of the model is justified, and the kind of overlapping curvature as shown in Fig S1(D) and (E), where the tip cells in the model cut the corners of a straight-edged astrocyte pre-pattern is realistic.

For older vessels this is not however the case, as the astrocytes adapt their morphology to match the endothelial cells, as shown in Fig. S1(F) and (G). Initially, tip cells follow the astrocyte road map, but may cut corners where there are holes due to membrane tension, whereupon the astrocytes adapt and support these overlaps from beneath.

Actin deactivates when receptors are no longer active. Based on an equilibrium constant determined experimentally [1], the profilin ATP G-actin complex dissociation rate has been shown to be 0.6 s-1. However, the actin token disassociation parameter (TokDis), as it is a mesoscale parameter for a memAgent, does not just reflect this dissociation rate. The rate for profilin to diffuse into the area for binding ATP-G actin also needs to be taken into account, thus the TokDis rate parameter represents the total time it takes for the current amount of profilin to disperse, leaving no active actin in the local cell region under consideration. TokDis was set to 5 timesteps in the model, estimating 75 seconds for this process. In our model, an activated receptor is required to extend a filopodia, so accumulation of actin alone does not generate unwanted filopodia, modelling the important role of WASP and proline rich proteins to the site of receptor activation for actin polymerisation to take place [2].

References

[1] Selden L A, Kinosian J H, Estes J E, Gershman L C (1999) Impact of profilin on actin-bound nucleotide exchange and actin polymerization dynamics. Biochemistry 38: 2769-2778.

[2] Holt M R, Koffer A (2001) Cell motility: proline-rich proteins promote protrusions. TRENDS in Cell Biology 11: 38-46.
