# Supplementary material for: Tipping the Balance: Robustness of Tip Cell Selection, Migration and Fusion in Angiogenesis
Source: PLoS Comput Biol. 2009 Oct 30;5(10):e1000549. doi: 10.1371/journal.pcbi.1000549 (PMC2762315; doi:10.1371/journal.pcbi.1000549)
Supplement: Text S3 — Voxelisation method. (0.02 MB PDF) [file pcbi.1000549.s010.pdf]

voxelisation

The voxelisation algorithm used is based on that in [1]. Initially every spring in the node mesh, excluding filopodia springs, is labelled as either right or left and horizontal or vertical. Regardless of whether this orientation changes during mesh stretching these definitions of springs remain, giving a permanent description of which node is connected to which. We use the following notation for the four connected nodes of a given node N, $N_{rh}, N_{lh}, N_{rv}, N_{lv}$, see Fig. S2(A).

Each mesh square is divided into two triangles. We define the corners of the two triangles as follows: given a node N, the upper triangle is defined by the points N, N_{rh}, N_{rh-rv} and the lower triangle by N, N_{rv}, N_{rv-rh}, see Fig. S2(B). For every grid site in the range of the triangles vertices, a two stage test is performed to see if the triangle intersects that grid site cube. For efficiency, as surface agents are only created to complete the mesh into a surface without holes, this is only performed if the grid site is currently not occupied by a memAgent from the same cell.

The first stage of the cubic-grid site and triangle intersection test checks whether any of the triangle lines intersect any of the cube face planes. To do this, the equation of the plane for each cube face is found and the point of intersection with each triangle line is calculated. If this point lies within the line segment, and the face plane bounded by the cube vertices, then the triangle is said to intersect that grid site and a new surface memAgent is created within it. If no line-face plane intersection can be found then the second stage of the test checks whether any diagonals within the cubic grid site intersect the plane of the triangle. For this the equation of each diagonal is found and the equation of the plane of the triangle. Once again the point of intersection is calculated and if it lies within the triangle boundaries and the grid site then a new surface memAgent is created in that grid site. See Figs. S3, S4 and S5.

References

[1] Voorhies D (1992) Graphics Gems III. Academic Press Professiona.
