# Supplementary material for: Tipping the Balance: Robustness of Tip Cell Selection, Migration and Fusion in Angiogenesis
Source: PLoS Comput Biol. 2009 Oct 30;5(10):e1000549. doi: 10.1371/journal.pcbi.1000549 (PMC2762315; doi:10.1371/journal.pcbi.1000549)
Supplement: Text S4 — Live imaging and simulation method specifics for quantification of the contact-veil advance filopodia mechanism. (0.03 MB PDF) [file pcbi.1000549.s011.pdf]

Live imaging and simulation method specifics for quantification of the contact-veil advance filopodia mechanism

Zebrafish Live Microscopy: $Tg(fli1 : EGFP)_{y1}$ transgenic line [1] were maintained at standard conditions as previously described [2]. Embryos at 28hpf were anaesthetized in systems water containing tricane (0.016 %, pH 7) and immobilized in 0.2% agarose on glass bottom culture dishes (MatTek Corp.). Embryos were kept at 28.0° in environmental chamber. Time-lapse microscopy was carried out on Nikon Eclipse TE200-U using Perkin Elmer UltraView ERS spinning disk system, 60X lens. EGFP was excited with 488nm laser emission supplied by an Argon laser. Stacks are composed of optical slices with 0.5mm slice spacing. Stacks were acquired every 2mins. Time-lapse video is a 2D representation of projected stacks.

Quantification of time-lapse videos: Videos were processed using Volocity (Improvision). Number and duration of contacts (see Fig S6 for example video frames and Fig. S7 for an example image of a contact between two filopodia) were quantified by observing the optical slices involved in event of any filopodia pair that appears to touch in a two-dimensional projection. Fusion events were determined by the stabilization of diameter of contact site where anastomosis has occurred.

In simulations the vessel section was comprised of just four cells. The unbiased astrocyte square lattice was used, the environment contained a background linearly increasing gradient of VEGF and a point source of VEGF midway between the second and fourth cells. In equation (3) in the main text : $v = 0.12, m = 0.1$ and $d = 30$. Outlier runs, where the first and third cells (from the left) were selected as tip cells, were discounted as they were not near enough to the point source to fuse. The filopodia contact information was gathered until the two tip cells fused. A contact was defined in the model as the event where one agent in a filopodia is located in the same grid site as an agent in a filopodia from a different cell. Contacts were characterised by the two filopodia involved, uniquely identified by the agent at the base, as long as those two filopodia had memAgents in contact the lifetime of the contact would be incremented. Therefore, contacts were not location specific, but filopodia specific, as it appears from live imaging data that contacts can slide along filopodia, or both filopodia may move in space.

References

[1] Lawson N D, Weinstein B M (2002) In vivo imaging of embryonic vascular development using transgenic zebrafish. Developmental Biology 248: 307-318.

[2] Kimmel C B, Ballard W W, Kimmel S R, Ullmann B, Schilling T F (1995) Stages of embryonic development of the zebrafish. Developmental Dynamics 203: 255-310.
